# MBD2a–NuRD binds to the methylated γ-globin gene promoter and uniquely forms a complex required for silencing of HbF expression

**DOI:** 10.1073/pnas.2302254120

**Published:** 2023-06-12

**Authors:** Shengzhe Shang, Xia Li, Alexander Azzo, Tin Truong, Mikhail Dozmorov, Charles Lyons, Asit K. Manna, David C. Williams, Gordon D. Ginder

**Affiliations:** ^a^Massey Cancer Center, Virginia Commonwealth University, Richmond, VA 23060; ^b^Department of Human and Molecular Genetics, Virginia Commonwealth University, Richmond, VA 23060; ^c^Center for Clinical and Translational Research, PhD Program in Cancer and Molecular Medicine, Virginia Commonwealth University, Richmond, VA 23060; ^d^MD-PhD Program, Virginia Commonwealth University, Richmond, VA 23060; ^e^Department of Biostatistics, Virginia Commonwealth University, Richmond, VA 23060; ^f^Department of Pathology and Laboratory Medicine, University of North Carolina, Chapel Hill, NC 27599; ^g^Department of Internal Medicine, Division of Hematology-Oncology, Virginia Commonwealth University, Richmond, VA 23060

**Keywords:** MBD2a–NuRD, fetal hemoglobin, BCL11A, DNA methylation, PRMT5

## Abstract

During human development, there is a switch in the erythroid compartment at birth that results in silencing of expression of fetal hemoglobin (HbF). Reversal of this silencing has been shown to be effective in overcoming the pathophysiologic defect in sickle cell anemia. Among the many transcription factors and epigenetic effectors that are known to mediate HbF silencing, two of the most potent are BCL11A and MBD2–NuRD. In this report, we present direct evidence that MBD2–NuRD occupies the γ-globin gene promoter in adult erythroid cells and positions a nucleosome there that results in a closed chromatin conformation that prevents binding of the transcriptional activator, NF-Y. We show that the specific isoform, MBD2a, is required for the formation and stable occupancy of this repressor complex that includes BCL11A, MBD2a–NuRD, and the arginine methyltransferase, PRMT5. The methyl cytosine binding preference and the arginine-rich (GR) domain of MBD2a are required for high affinity binding to methylated γ-globin gene proximal promoter DNA sequences. Mutation of the methyl cytosine–binding domain (MBD) of MBD2 results in a variable but consistent loss of γ-globin gene silencing, in support of the importance of promoter methylation. The GR domain of MBD2a is also required for recruitment of PRMT5, which in turn results in placement of the repressive chromatin mark H3K8me2s at the promoter. These findings support a unified model that integrates the respective roles of BCL11A, MBD2a–NuRD, PRMT5, and DNA methylation in HbF silencing.

## Significance

Reversal of fetal hemoglobin (HbF) silencing can ameliorate the effects of sickle cell anemia. Despite available gene therapy and stem cell transplantation modalities, the majority of affected patients worldwide will not have access to these in the near future. Thus, there is a need for safe and effective small-molecule therapeutics. We report here that stable occupancy of a major HbF silencing complex containing BCL11A, MBD2a–NURD, and PRMT5 and exclusion of the transcriptional activator NF-Y at the γ-globin gene promoter require specific features of MBD2a. These results provide a unified model for the relationships between the previously reported HbF silencers MBD2–NuRD, BCL11A, DNA methylation, and PRMT5 that may facilitate development of therapeutic agents to reverse HbF silencing.

The study of vertebrate globin gene switching during erythroid development has provided many primary insights into higher eukaryotic gene regulation. In humans, the switch in the β-globin gene locus involves sequential activation followed by silencing of the ε- and γ-globin genes (HBG1 and HBG2) during embryonic and fetal erythroid stages, respectively, resulting in predominantly β-globin gene expression, and hence hemoglobin A (HbA), shortly after birth. A number of known transcription factors are critical for mediating silencing of the fetal γ-globin genes in adult human erythroid cells, including BCL11A, LRF/ZBTB7A, EKLF/KLF1, and ZNF410, among others (1–5). In addition, several epigenetic factors including MBD2–NuRD (nucleosome remodeling and deacetylase) have been shown to mediate fetal globin gene silencing (6–8). In the case of the transcription factors BCL11A, LRF/ZBTB7A, and ZNF410, there is a direct interaction or relationship between each of these and a NuRD complex or one of its components (4, 5, 7).

The role of MBD2 and its associated NuRD corepressor complex in embryonic/fetal β-type globin gene silencing has been established in our previous studies in both transgenic animal and human erythroid cell models. In the transgenic animal model in which an entire human β-globin locus was inserted, this effect appeared to be largely dependent on DNA methylation (6, 7, 9, 10). However, the mechanistic explanation for the observation that MBD2–NuRD but not MBD3–NuRD is required for γ-globin gene (HBG1/2) silencing (10, 11) and direct evidence for MBD2–NuRD occupancy at the HBG promoters in adult human erythroid cells have been lacking. Genome-wide ChIP-seq analysis has confirmed gene-specific data showing that MBD2 binds with strong preference for so-called methylated CpG islands consisting of DNA containing 10 to 12 methylated CpG dinucleotides within 200 bp (12). In contrast to the CpG-rich avian embryonic ρ-globin gene promoter, to which MBD2 was shown to bind directly in definitive erythroid cells in which the gene is silenced (7), the human HBG promoters do not contain highly CpG-rich DNA sequences. However, in vitro binding studies have shown that MBD2 or its methyl-cytosine-binding domain can bind preferentially to DNA fragments with as few as 1 to 2 methyl-CpG dinucleotides (13). In addition, various inhibitors of DNA methylation induce increased HbF in adult primate animal models and in patients with hemoglobinopathies (14, 15).

In an effort to elucidate the basis for the observation that the MBD2–NuRD complex and not the MBD3–NuRD complex is required for full silencing of the γ-globin gene in adult human erythroid cells, studies of its occupancy at the promoter and its effects on nucleosome positioning and chromatin structure, as well as its interactions with other known silencing factors, were performed. Our findings indicate that MBD2 occupies the methylated HBG promoters in adult erythroid cells in which these gene are silenced and support the concept that MBD2 and BCL11A act as cofactors in the same silencing complex. These studies reveal that the MBD2a isoform is more capable than MBD2b in mediating γ-globin silencing due to a combination of the preference of its methyl cytosine–binding domain (MBD) for the methylated HBG promoters and its N-terminal arginine–rich GR domain, which together impart high-affinity binding to methylated promoter DNA. In addition, the GR domain recruits MEP50/PRMT5 to the promoter, which contributes to a closed chromatin conformation and silencing.

## Results

### MBD2–NuRD Occupies the HBG Promoter in Adult Erythroid Cells and Positions a Nucleosome That Enforces a Closed Chromatin Configuration.

**Fig. 1.**
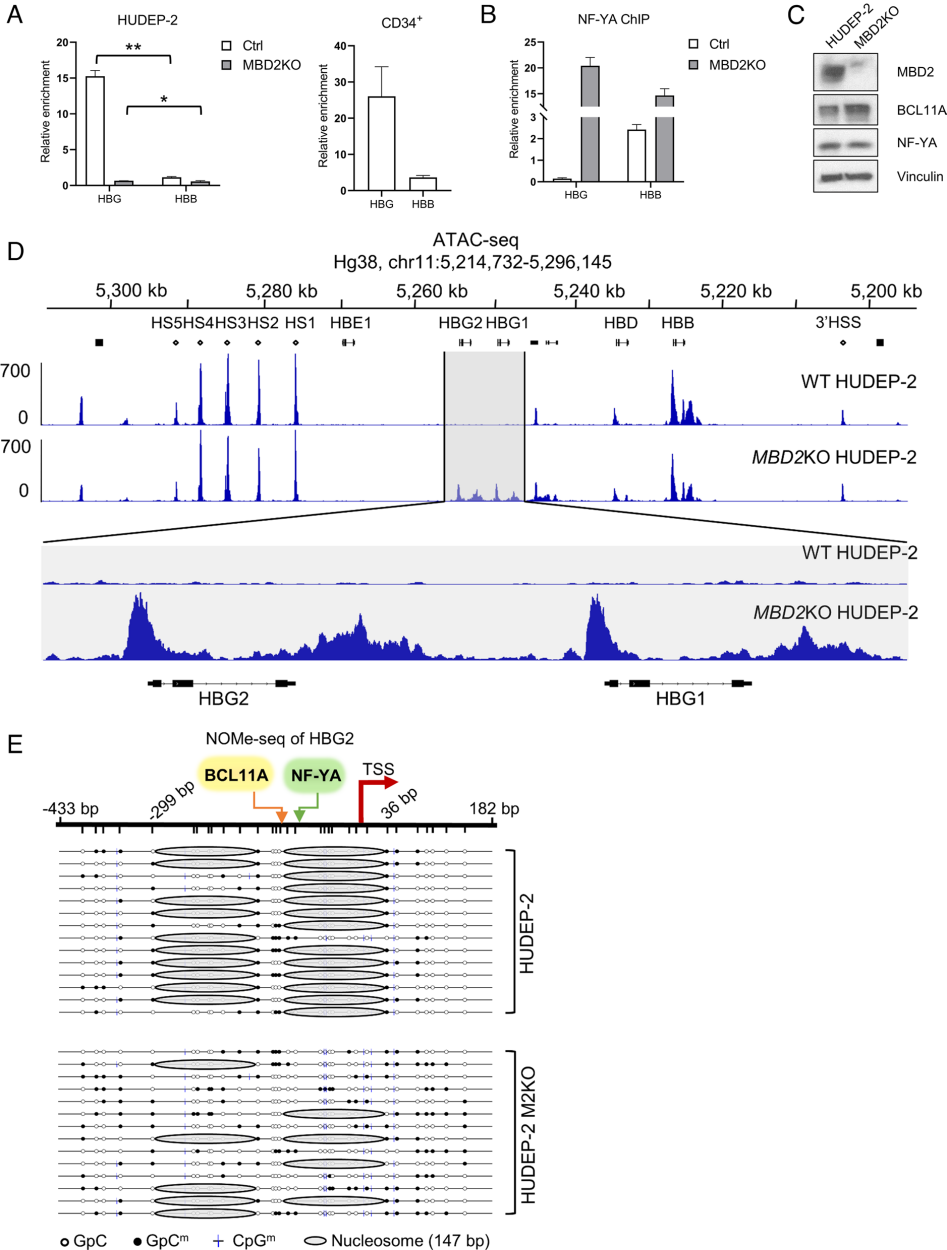
MBD2–NuRD occupies the HBG gene promoter in HUDEP-2 cells and CD34+ cells and positions a nucleosome that enforces a closed chromatin configuration to block NF-Y binding at the HBG promoters. (*A*) ChIP-qPCR assay shows that MBD2 occupies the HBG promoter but not β-globin (HBB) gene promoter in HUDEP-2 cells (mean ± SD, *N *= 2) and CD34^+^ cells. The CD34^+^ ChIP result is shown as the mean ± SD of three technical repeats. *P < 0.05, ** *P *< 0.01. (*B*) ChIP-qPCR assay shows that NF-YA binds at the HBG gene promoter in differentiated parental and M2KO HUDEP-2 cells. The result is shown as the mean ± SD of three technical repeats and is representative of two biological repeats. (*C*) Protein level of NF-YA and BCL11A in parental and M2KO HUDEP-2 cells. (*D*) ATAC-seq showing open chromatin at the HBG1 and HBG2 genes in MBD2KO HUDEP-2 cells. (*E*) NOMe-seq showing a fixed nucleosome at position −110 to +36 bp relative to the transcription initiation site (TSS) in parental HUDEP-2 cells that is evicted in MBD2KO cells.

ChIP-qPCR assays (as shown in Fig. 1 and *SI Appendix, *Table S1) in the HUDEP-2 erythroid cell line (16) and primary CD34^+^ progenitor cells demonstrated direct binding of MBD2 to the human HBG promoter (Fig. 1*A*). The CCAAT box binding complex, NF-Y, has been shown to be a major transcriptional activator of the HBG genes that is displaced in the presence of high levels of BCL11A (17). ChIP-qPCR assays for NF-YA, the sequence-specific binding component of the NF-Y CCATT box binding complex, showed that it is bound to the HBG promoters in MBD2 KO cells despite an increase in the level of BCL11A (Fig. 1 *B* and *C*). ATAC-seq assays in parental and MBD2KO HUDEP-2 cells (Fig. 1*D*) showed equivalent chromatin opening at the two HBG promoters HBG1 and HBG2, as was observed in BCL11A knockout cells (18). To further elucidate the mechanism through which MBD2–NuRD enforces γ-globin gene silencing and exclusion of NF-YA, we carried out Nucleosome Occupancy and Methylome Sequencing (NOMe-seq) analysis in both parental and MBD2KO HUDEP-2 cells. As shown in Fig. 1*E*, there is a nucleosome fixed at nucleotide position −110 to +36 relative to the transcription start site (TSS) of the HBG2 promoter in wild-type parental cells, thus occluding the preferred binding sites for NF-YA at the nucleotide position −89 relative to the TSS and for the transcription initiation complex of the HBG genes, but not the preferred binding site for BCL11A centered at the nucleotide position −115, which has been shown by Liu et al to occupy this site in wild-type parental HUDEP-2 cells (17). Occupancy of this nucleosome is significantly decreased in MBD2KO HUDEP-2 cells compared to parental HUDEP-2 cells, allowing NF-YA to bind at the HBG promoter (Fig. 1*B*), despite an increase in the level of BCL11A protein and no change in NF-YA protein level (Fig. 1*C*). These data demonstrate the critical role of MBD2–NuRD in establishing a closed chromatin configuration at the HBG promoters in adult cells that excludes binding of the major transcriptional activator complex NF-Y and the transcription initiation complex of these genes. In addition to the identical ATAC-seq data for both HBG1 and HBG2 genes, LC–MS quantitation showed equal levels of both corresponding γ-globin chains (*SI Appendix,* Fig. S1), in parental and MBD2KO cells, consistent with the same nucleosome positioning at both loci.

### The GR Domain and Methylated CpG Preference of the MBD2a MBD Are Required for High-Affinity Binding to Methylated Proximal HBG Promoter Sequences.

**Fig. 2.**
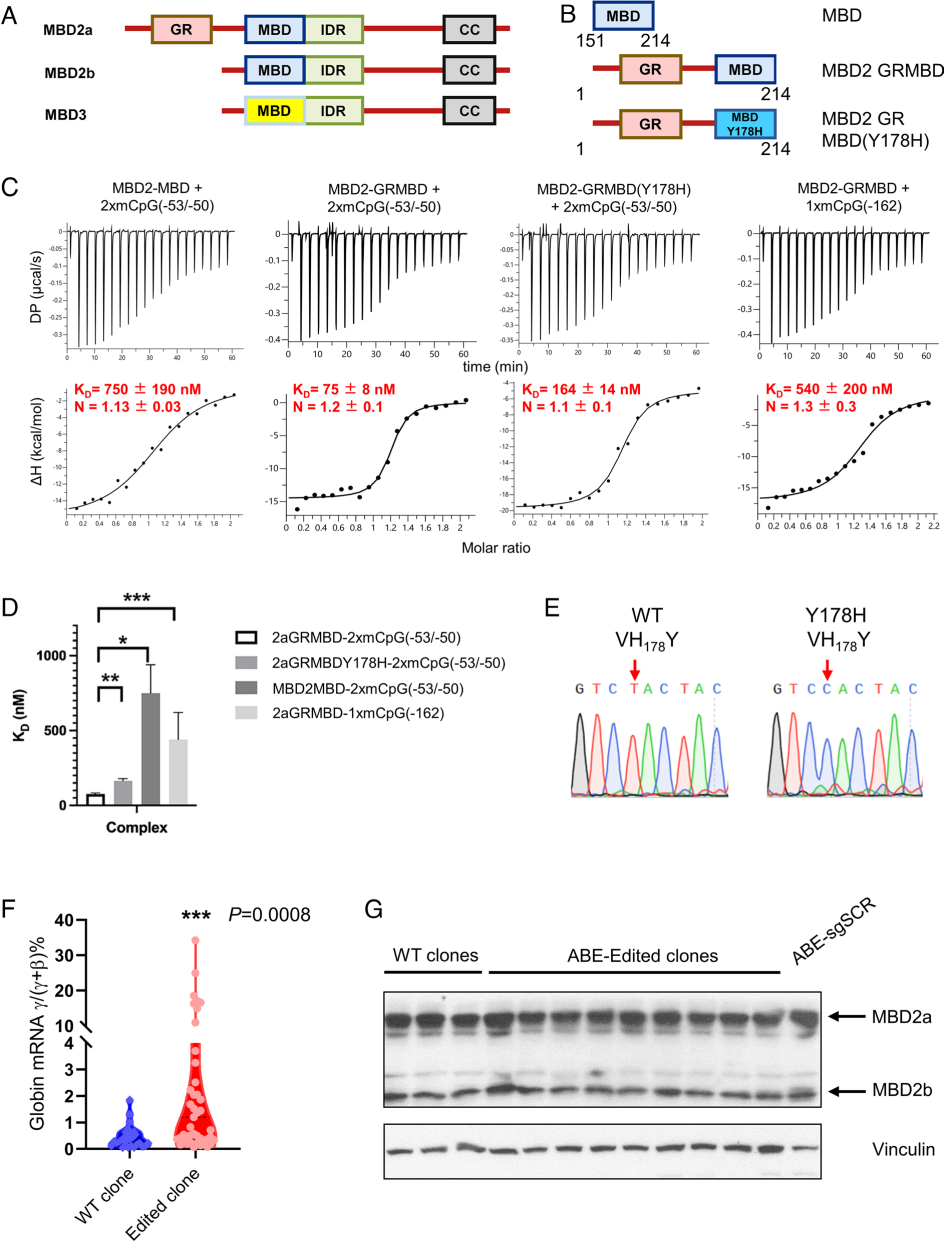
DNA methylation and the GR domain enhance the binding of the MBD2 methylcytosine–binding domain to the γ-globin promoter DNA sequences. (*A*) Schematic representation of the protein structure of MBD2a vs. MBD2b and MBD3. (*B*) Schematic illustration of the structure of the peptides used in binding affinity studies: MBD2-MBD, GRMBD, and GRYMBD(Y178H). (*C*) Binding affinity plots for MBD2-MBD, MBD2-GRMBD, and MBD2- GRMBD(Y178H) binding to methylated DNA encompassing the proximal [2xmCpG (−53/−50)] and distal [1xmCpG (−162)] CpG sites of the HBG promoters were determined by isothermal titration calorimetry. The K_D_ and N values represent the mean ± SD for three to four replicates. (*D*) A bar graph depicts the K_D_ for each of the four complexes, compared using Welch’s unpaired *t* test. (*E*) Sanger sequencing result showing the specific adenine base editing to introduce the Y178H mutation in endogenous MBD2 genomic DNA. In wild-type clones, the amino acid sequence is _177_VYY_179_. In edited clones, the Y178 is changed to H, the amino acid sequence is _177_VHY_199_. The edited base is indicated by red arrow. (*F*) Q-PCR results showing the γ/(γ+β) mRNA ratio in ABE8e-MBD2 Y178–edited HUDEP-2 cell single colonies. *P* value was calculated by Welch’s unpaired *t* test. Blue dots represent the scramble guide clones without editing at MBD2Y178 (*N *= 26), red dots represent the clones with editing at MBD2Y178H amino acid position (*N *= 47). (*G*) Immunoblotting panel showing equivalent levels of total MBD2 protein in edited single clones and scramble guide control clones. *P < 0.05, **P < 0.01, ***P < 0.001.

MBD2 differs from MBD3 in that the former has a much higher affinity for DNA-containing methylated CpG dinucleotides (Fig. 2*A*) (19). MBD2a and MBD2b are the two major isoforms of MBD2 found in somatic cells. They differ by the presence of an arginine-rich (GR) domain found only in MBD2a that results from an alternate upstream ATG translation start site (*SI Appendix*, Fig. S2).

Although, as noted, there is no CpG island in the HBG promoter, there are six CpG dinucleotides within the first 200 nucleotides of the HBG promoter and first exon. Five of these are located in sequences from −53 bp upstream to +46 bp downstream relative to the TSS and are nearly fully methylated in both the adult phenotype HUDEP-2 proerythroblast cell line and adult human CD34^+^ progenitor–derived primary erythroid cells (*SI Appendix*, Fig. S3). In order to determine the potential roles of the MBD2-binding domain and the GR domain in HBG silencing, in vitro binding studies were carried out with methylated HBG promoter sequences containing two methylated CpG sites at positions −53 and −50 relative to the TSS. For these studies, isothermal calorimetry assays were performed with peptides containing the MBD2 MBD with or without the GR domain. Additional assays were carried out with a peptide containing the GR domain and an MBD2 MBD domain with a Y178H mutation, which decreases its binding preference for methylated cytosines (Fig. 2*B*).

As shown in Fig. 2 *C* and *D* and *SI Appendix,* Fig. S4, there was a very low binding affinity for the MBD alone (K_D_ = 750 ± 190 nM). As expected, the MBD does not bind to unmethylated DNA, consistent with the 100-fold selectivity for methylated DNA that was reported previously (*SI Appendix,* Fig. S5*C*) (20–23). By far, the highest binding affinity was seen with the peptide containing a wild-type MBD plus the GR domain (K_D_ = 75 ± 8 nM). Hence, the GR domain increases the affinity by 10-fold. In contrast, the binding affinity of the GR domain plus wild-type MBD peptide for methylated promoter sequences containing the −162 methyl CpG (K_D_ = 540 ± 200 nM) was much lower than for the −53/−50 CpG–containing sequences (Fig. 2*C*). Notably, MBD2a binds to either sequence with a stoichiometry of 1:1 (N ~ 1) despite the presence of two methylated CpGs in the −53/−50 CpG oligonucleotide. As expected from the structure of MBD2-MBD bound to DNA (20), the proximity of these CpGs prevents two MBD2a proteins from binding simultaneously.

To test whether methylation selectivity of the MBD contributes to the high-affinity association and gene silencing, we mutated a tyrosine residue (Y178) that directly interacts with a methyl-cytosine residue and plays a central role in preferential binding to methylated DNA (20–24). We chose a mutation amenable to adenine base editing (Y178H) to enable introduction of the same mutation in HUDEP-2 cells. As expected, MBD2-MBD (Y178H) binds methylated DNA with an affinity reduced by approximately 100-fold (K_D_ = 77,000 ± 22,000 nM, *SI Appendix*, Fig. S5*A*). At the same concentrations that we used for wild-type protein, MBD2-MBD (Y178H) did not bind unmethylated DNA, but at higher concentrations, we could detect very weak binding (K_D_ = 344,000 nM, *SI Appendix*, Fig. S5*B*), compatible with approximately fivefold selectivity for methylated DNA. This selectivity is much lower than for the unmutated domain and is comparable to the effects of the Y178F mutation studied previously (21, 23). To test whether this mutation impacts binding in the context of the GR domain, we introduced the Y178H mutation into the construct that contains both the GR and MBD domains. As shown in Fig. 2 *C* and *D*, the MBD2-GRMBD(Y178H) shows an approximately twofold reduction in binding affinity (K_D_ = 164 ± 14 nM), indicating that the GR domain partially compensates for the Y178H mutation.

### The Preferential Binding of MBD2 to Methylated DNA Sequences of the HBG Promoter Contributes to Silencing.

Bisulfite sequencing was performed which confirmed that all of the CpG sites in the HBG proximal promoter are fully methylated in parental HUDEP-2 cells (*SI Appendix*, Fig. S3*A*). To determine the importance of the methylated cytosine–binding preference of MBD2a for binding to the promoter and silencing, site-specific base editing of the endogenous MBD2 MBD was carried out in HUDEP-2 cells to introduce the Y178H mutation in the MBD2 MBD domain (Fig. 2*E*, and *SI Appendix,* Fig. S6*A* and Table S2). As shown in Fig. 2*F*, clones containing the Y178H mutation (*N *= 47) had elevated levels of the percentage of γ/(γ+β) RNA which is the result of an increased γ-globin RNA level (*SI Appendix,* Fig. S6 *C* and *D*), compared with wild-type control cell clones (*N *= 26). The effect was highly statistically significant but variable, with the percentage of γ/(γ+β) mRNA levels ranging from 0.13% to 34.24% compared to a range from 0.04% to 1.00% in control cell clones. The MBD2 protein level in wild-type clones and edited clones is equivalent to the level in ABE-sgSCR control HUDEP-2 cells (Fig. 2*G*).

We interpret the variability in the levels of γ-globin gene expression to reflect the fact that the Y178H mutation decreases the methylcytosine preference of the MBD, but does not completely negate it in the context of the GR domain of MBD2a. Rather, we postulate that it decreases the probability of MBD2a–NuRD localizing at the promoter and in turn establishing a repressive chromatin configuration there to block binding of NF-Y and the transcription initiation complex.

### NMR Analysis of the GR Domain Reveals a Largely Unstructured Domain, Even in the Presence of DNA.

To further assess the structure and DNA-binding properties of the GR domain, we collected 2D-^15^N-HSQC spectra of the domain in the presence of increasing concentrations of DNA. As shown in *SI Appendix,* Fig. S7*A*, the spectrum of the isolated domain shows typical characteristics of an unstructured protein, with very intense and narrow peaks, lack of chemical shift dispersion in the ^1^H dimension, and extensive spectral overlap. Adding DNA leads to relatively small chemical shift changes throughout the spectrum without significant line-broadening or chemical shift dispersion and independent of DNA methylation (*SI Appendix*, Fig. S7 *A* and *B*). Notably, the chemical shifts do not follow a linear change based on DNA concentration. Instead, many affected peaks shift in different directions as the protein concentration exceeds that of the DNA. This observation suggests that when in excess, more than one GR domain weakly interacts with the same DNA.

We then compared 2D-^15^N-HSQC spectra for the isolated MBD and GR-MBD proteins bound to the same methylated DNA. As shown in *SI Appendix,* Fig. S7*C*, the GR domain shows characteristics of an unstructured domain even in the presence of the MBD and when bound to DNA. The chemical shifts are essentially identical to that of the isolated domain. Likewise, the MBD shows similar chemical shifts when bound to DNA with or without the GR domain (*SI Appendix*, Fig. S7*D*). These data indicate that the GR domain behaves as a fuzzy DNA–binding region, promoting DNA binding without adopting a regular structure. Our previous studies showed that the central intrinsically disordered region of MBD2 behaves similarly (25). Fuzzy protein–DNA interactions involving protein regions adjacent to a DNA-binding domain, and contributing to DNA-binding affinity through transient interactions, are increasingly recognized (26).

### The GR Domain of MBD2a Is Required for Silencing and Recruits MEP50/PRMT5.

**Fig. 3.**
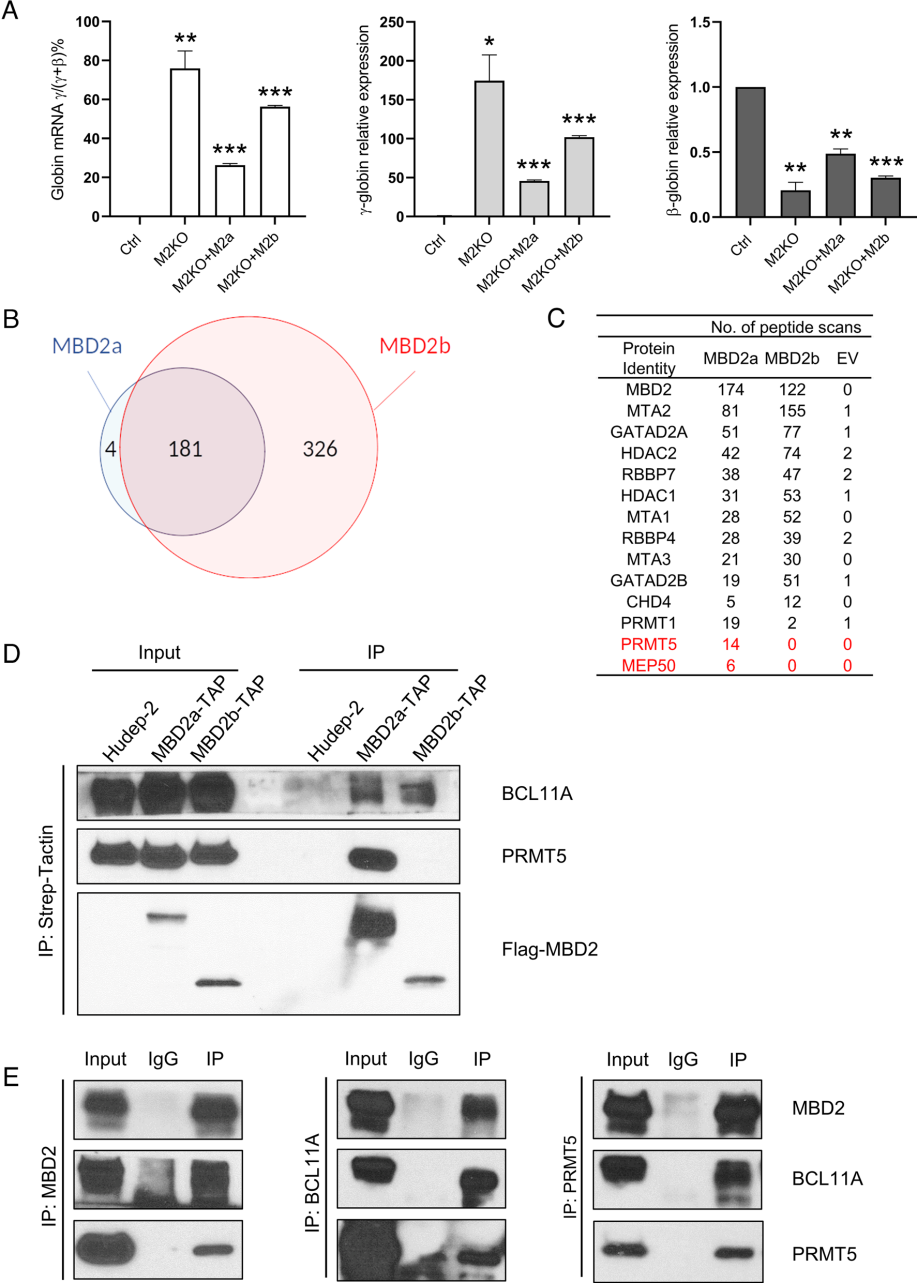
MBD2a–NuRD and not MBD2b–NuRD specifically associates with both BCL11A and PRMT5. (*A*) Q-PCR in differentiated parental or MBD2KO HUDEP-2 cells with MBD2a versus MBD2b-TAPtag addback shows that MBD2a but not MBD2b enforces γ-globin gene repression. *P* value is with respect to control (mean ± SD, *N *= 2, the *P* value was calculated by the Student’s *t* test. *P < 0.05, **P < 0.01, ***P < 0.001). (*B*) Venn diagram of unbiased subtraction proteomic analysis of nuclear extract proteins immunoprecipitated with MBD2a versus MBD2b TAP-tagged bait. (*C*) Peptide enumeration showing the presence of core NuRD components in both MBD2a and MBD2b immunoprecipitated protein samples and differential association of PRMT5/MEP50 peptides only in the MBD2a immunoprecipitation. The same result was obtained with the corresponding whole-cell extract. EV represents empty vector control. (*D*) Strep-Tactin immunoprecipitation western blot assay showing only MBD2a-TAPtag associated with both BCL11A and PRMT5. (*E*) Reciprocal immunoprecipitation of endogenous MBD2, BCL11A, and PRMT5 proteins in parental HUDEP-2 cells showing mutual association of all the three proteins. The immunoprecipitation results shown are representative of three independent biological repeats.

In order to determine the degree of γ-globin gene silencing mediated by MBD2a and MBD2b, enforced expression of each MBD2 individually was carried out in MBD2KO HUDEP-2 cells. As shown in Fig. 3*A*, MBD2a expression was able to silence the γ-globin gene expression significantly more than MBD2b. The degree of repression observed is less than that present in wild-type HUDEP-2 cells. We attribute this to presumed spatiotemporal differences in the activity of MBD2 when its expression is driven by an exogenous promoter construct compared to its endogenous activity, as we have previously observed (10).

Further studies were carried out to investigate whether or not the GR domain recruits additional factors involved in γ-globin gene silencing in addition to its role in increasing the binding affinity of MBD2a for methylated HBG promoter sequences. Unbiased subtraction proteomic analysis was performed on nuclear proteins immunoprecipitated by enforced expression of TAP-tagged MBD2a or MBD2b in MBD2KO HUDEP-2 cells (*SI Appendix*, Fig. S2*B*) followed by affinity purification. As shown in Fig. 3 *B* and *C*, only four peptides were uniquely associated with MBD2a. Of these, PRMT5 and MEP50 were of particular interest due to the previous implication of PRMT5 in γ-globin gene silencing (27). MEP50/PRMT5 is an obligate hetero-octameric complex that catalyzes symmetric dimethylation of arginine residues (28). Deletion mapping of the MBD2a construct in both the functional addback assay and immunoprecipitation assays demonstrated that the N-terminal 72 amino acids of the GR domain were required for silencing (*SI Appendix*, Fig. S8).

Studies of BCL11A have demonstrated its association with the MBD3–NuRD complex in murine erythroid cells (29). Results reported by us and others (8, 10, 11) demonstrated that MBD2–NuRD but not MBD3–NuRD is required for full silencing of the γ-globin genes in human HUDEP-2 and CD34^+^ cells. As shown in Fig. 3 *D* and *E*, immunoprecipitation experiments demonstrated that only MBD2a and not MBD2b associates with both BCL11A and PRMT5 in HUDEP-2 cells. Previous studies from our lab and others showed that knockout of either BCL11A or MBD2 in HUDEP-2 cells and knockdown of either in CD34^+^ cells result in very similar levels of transcriptional activation of the HBG genes (1, 3, 8, 10), further supporting the concept that these two factors act in conjunction to enforce silencing.

### MEP50/PRMT5 Occupancy at the HBG Promoter Depends on MBD2–NuRD Occupancy and Contributes to Silencing.

**Fig. 4.**
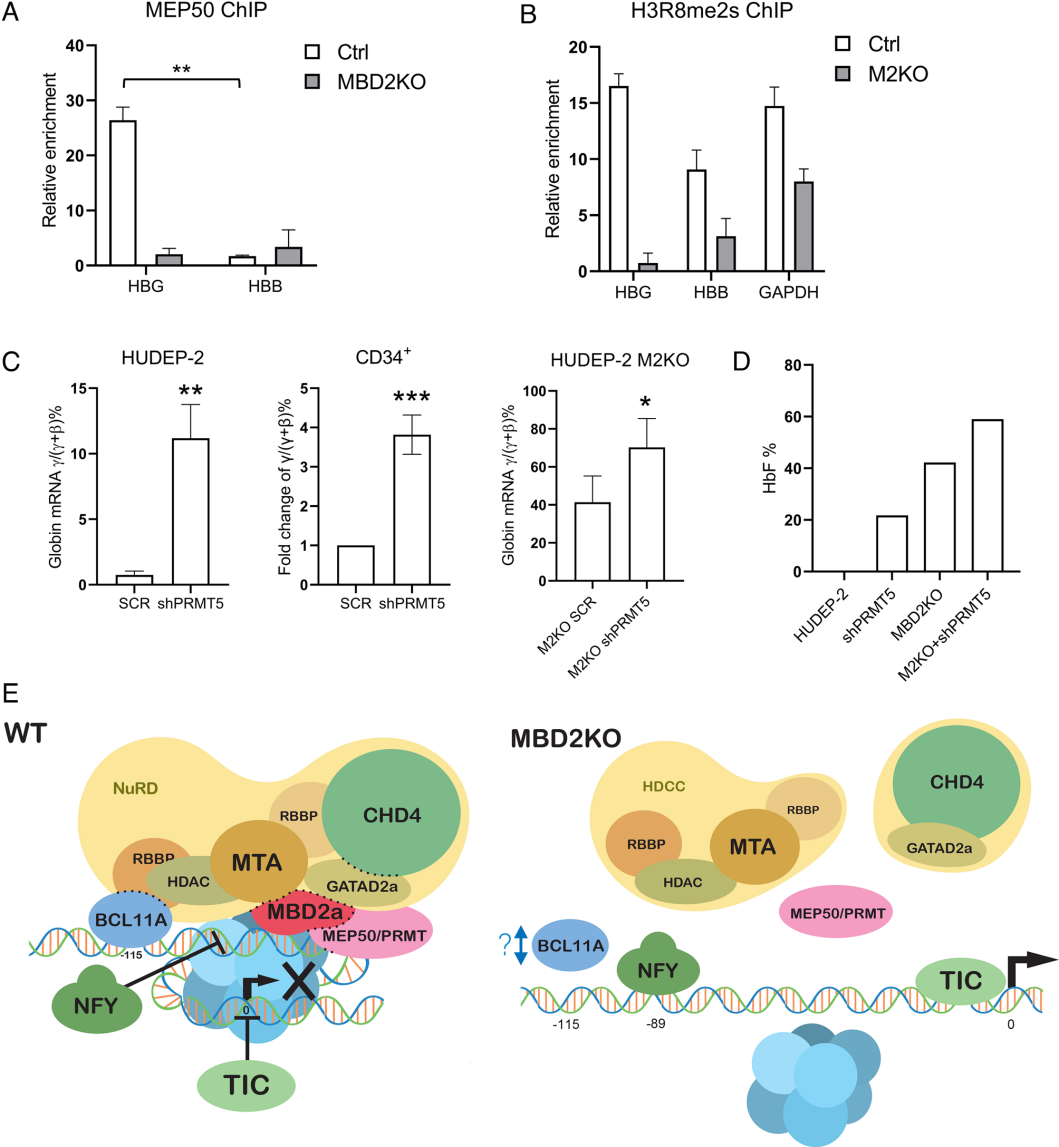
MBD2a recruits PRMT5/MEP50 to the promoter which contributes to γ-globin silencing. (*A*) MEP50 ChIP-qPCR results showing MEP50/PRMT5 enrichment at the γ-globin promoter in parental HUDEP-2 cells but not in MBD2KO cells (mean ± SD, *N *= 2). *P < 0.05, **P<0.01. (*B*) ChIP-qPCR assay of histone H3R8me2s occupancy showing that the repressive marker is more enriched at the HBG promoter in parental vs MBD2KO HUDEP-2 cells. The result is shown as the mean ± SD of three technical repeats and is representative of two biological repeats. (*C*) Q-PCR assay of γ/(γ+β) mRNA ratio in PRMT5KD of both HUDEP-2 cells (mean ± SD, *N *= 3) and CD34+ progenitor cells (mean ± SD, *N *= 3) and MBD2KO of HUDEP-2 cells (mean ± SD, *N *= 5). *P < 0.05, **P < 0.01, ***P < 0.001. (*D*) LC–MS analysis of globin chains showing the relative Hb percent of total hemoglobin in parental, PRMT5-depleted, MBD2KO, and M2KO plus PRMT5-depleted HUDEP-2 cells. (*E*) Schematic illustration of the HBG gene corepressor complex model showing the requirement for MBD2a–NuRD for maintenance of a positioned nucleosome at the HBG promoter to prevent binding of the transcriptional activator, NF-Y, and the MBD2a-dependent cooccupancy of PRMT5 in the complex in wild-type HUDEP-2 cells. In MBD2a knockout cells, the MBD2a–NuRD complex along with PRMT5 is dissociated from the promoter, the nucleosome is evicted, and the NF-Y complex binds at the CCAAT box at position −89 relative to the TSS to activate transcription.

To determine whether MEP50/PRMT5 occupies the HBG promoter in association with MBD2–NuRD, MEP50 ChIP-qPCR analysis was carried out with parental and MBD2KO HUDEP-2 cells. The ChIP-qPCR assay showed that MEP50, the obligate binding partner of PRMT5, occupied the HBG promoter in parental HUDEP-2 cells, but not in MBD2KO cells (Fig. 4*A* and *SI Appendix,* Table S1). As histone H3R8 symmetric dimethylation is considered a repressive mark for gene expression mediated by MEP50/PRMT5, we performed ChIP-qPCR assays of symmetrically dimethylated H3R8 histones (H3R8me2s), which showed high level of H3R8me2s occupancy at the HBG promoter in parental HUDEP-2 cells that was significantly diminished in MBD2 KO HUDEP-2 cells (Fig. 4*B*). To establish the functional role of MEP50/PRMT5 in gene silencing through the MBD2–NuRD complex, PRMT5 was depleted in both parental and MBD2KO HUDEP-2 cells, as well as in adult CD34^+^ progenitor–derived primary erythroid cells with knockdown efficiency greater than 90% and ~70%, respectively (*SI Appendix*, Fig. S9). PRMT5 knockdown (PRMT5KD) resulted in 20-fold and fourfold increases in γ-globin mRNA levels, respectively, in HUDEP-2 and CD34^+^ cells (Fig. 4*C*). In contrast, knockdown of PRMT5 in MBD2KO HUDEP-2 cells resulted in only a 1.5-fold further increase in γ-globin mRNA expression. This increase is minimal compared to the nearly 100-fold effect from MBD2 knockout alone. Consistent with the RNA assay results, PRMT5 depletion resulted in only a 0.5-fold increase in fetal hemoglobin level in MBD2 KO cells compared to the ~150-fold effect from MBD2 depletion alone (Fig. 4*C*). These results support the proposition that most of the silencing effect of PRMT5 is through its presence in the MBD2–NuRD complex.

## Discussion

The chromatin remodeling complex MBD2–NuRD and its components, which include CHD4, GATAD2A and histone deacetylases, have been shown to be essential for full γ-globin gene silencing (6–8, 10, 11, 29, 30). Likewise, the repressive transcription factor BCL11A plays a central role in developmental γ-globin gene silencing (1, 18, 31, 32). Depletion of either MBD2–NuRD or BCL11A results in equivalent levels of increased expression of HbF in both the immortalized adult human erythroid cell line, HUDEP-2, and primary CD34^+^ progenitor cells. Consistent with the physical interaction between BCL11A and NuRD (33), data presented here indicate that there is a direct interaction between MBD2–NuRD and BCL11A.

Although not without controversy, significant published evidence supports a role for epigenetic signals such as DNA methylation and histone modifications in silencing the expression of HbF in adult erythroid cells (14, 15, 27, 34–38). Despite evidence for the important roles of these mediators of HBG silencing, a unified model that accounts for their respective roles and interactions relative to other HBG repressors/negative regulators has been lacking. In the studies reported here, we have sought to elucidate why it is only MBD2–NuRD and not MBD3–NuRD that is required for silencing the HBG promoter. We specifically examined its relationship with BCL11A in excluding the binding of the transcriptional activator NF-Y at the promoter and how DNA methylation is involved.

The data presented here demonstrate that MBD2 is bound directly at the HBG promoter in both HUDEP-2 and primary CD34^+^–derived erythroid cells. Importantly, we show that the GR domain of MBD2 is critical for both MBD2 association with methylated proximal HBG promoter sequences and recruitment of MEP50/PRMT5. The latter complex imparts a repressive H3K8me2s histone mark at the promoter. Further, these studies show that a nucleosome is positioned on the promoter between nucleotide position −108 bp and +36 relative to the TSS in parental HUDEP-2 cells, but is displaced in MBD2 knockout cells. A nucleosome positioned over a promoter at a TSS has been shown to inhibit active transcription by restricting access of the transcription initiation complex (39–41). This can be overcome only by strong “pioneering” transcriptional activator factors (42).

It is notable that the nucleosome positioned at the promoter in parental HUDEP-2 cells, in which HbF expression is silent, is located in a position that would block access of both the key transcriptional activator complex NF-Y, whose preferred binding site is at nucleotide position −89 relative to the TSS, and the transcription initiation complex at the TSS, but would not block access for BCL11A to bind at its preferred binding site at nucleotide position −115. Moreover, the NF-YA DNA-binding domain is not of the type that has been shown to compete successfully with nucleosomes for DNA occupancy (41). Consistent with the role of this nucleosome in excluding NF-Y is the finding that the eviction of the nucleosome in MBD2KO HUDEP-2 is accompanied by gain of NF-Y occupancy at the HBG promoter, despite an increase in the amount of BCL11A protein in the knockout cells. These data support a model in which MBD2a–NuRD both facilitates and is necessary for the previously demonstrated competition between BCL11A and NF-Y for occupancy at the HBG promoter in the repressed state (17, 18). Based on the positioning of the nucleosome at the promoter by NOMe-seq analysis, the known BCL11A-binding site centered at −115, and the relative position of the high-affinity methylated binding site for MBD2 (−53/−50), we built a molecular model of BCL11A and MBD2 on a nucleosome. The BCL11A-binding site is on the linker DNA immediately adjacent to the nucleosome when the −53/−50 CpG sites are on the dyad of the nucleosome. Hence, this model raises the possibility that both BCL11A and MBD2A would be in close proximity if they occupied their respective binding sites on the positioned nucleosome of the silenced promoter (*SI Appendix*, Fig. S10).

MBD2 differs from MBD3 largely by the fact that its unique MBD imparts highly preferential binding to methylated CpG-rich DNA (19–21). Somatic cells contain two major isoforms of MBD2. MBD2a contains an GR amino terminal domain (GR domain). The other isoform, MBD2b, arising from an alternate upstream ATG translational initiation site, lacks the GR domain, as does MBD3 (43). Previous studies have shown that MBD2b has a greater ability to replace the function of MBD3 than does MBD2a in neural stem cells, confirming a distinct difference in the function of these two isoforms (44). Here, it is shown that when either MBD2a or MBD2b was individually expressed in MBD2KO HUDEP-2 cells, MBD2a resulted in much more effective repression of γ-globin gene expression than did MBD2b. These results point to the critical role of the combination of the GR domain and the MBD uniquely present in MBD2a.

Two approaches were taken to determine the contributions of the high-affinity methyl CpG–binding domain and the GR domain of MBD2a to HBG gene silencing. First, a series of detailed in vitro binding assays indicated that high-affinity binding of the MBD2-specific MBD to methylated HBG promoter sequences required the presence of the GR domain and a wild-type MBD since either absence of the GR domain or introduction of a Y178H amino acid substitution in the MBD resulted in decreased affinity. The functional importance of the wild-type MBD2 MBD for γ-globin gene silencing in adult erythroid cells was confirmed by editing the endogenous MBD2 gene in HUDEP-2 cells to introduce the same Y178H mutation that diminishes in vitro binding. This resulted in a highly statistically significant but variable increase in the percentage of γ/(γ+β) mRNA levels among individual edited clones, ranging from 0.1% to 34%. We interpret this variability to be reflective of the fact that the Y178H mutation only modestly reduces the binding affinity of MBD2-GRMBD region. This suggests that promoter methylation is required for the highest binding affinity, and thus highest probability of MBD2a–NuRD occupancy at the HBG promoter, which in turn is required for complete silencing. Since there is a relative paucity of methylated CpGs in the promoter compared to CpG islands, it seems likely that stable occupancy of the MBD2a–NuRD complex requires binding of BCL11A, as reflected by the same degree and pattern of chromatin opening and increased level of HBG transcription when either is depleted (10, 18). The phenomenon of a requirement for multiple factors in cooperation to stabilize DNA binding that results in regulation of gene expression has been well documented (45).

To further investigate the role of the MBD2a GR domain, unbiased proteomic analysis of MBD2a–NuRD vs. MBD2b–NuRD complexes from HUDEP-2 cells revealed that only four proteins were uniquely associated with MBD2a. One of these, PRMT5, has been previously implicated in HbF silencing (27). PRMT5 is also important in MBD2–NuRD-associated gene silencing in tumor cells through PRMT5-mediated symmetric methylation of histone arginine residues and is associated with MBD2a but not MBD2b in HeLa cell extracts (46). We observed occupancy by the obligate binding complex MEP50/PRMT5 at the HBG promoter in parental but not MBD2KO cells. Thus, MEP50/PRMT5 occupancy is dependent on the presence of MBD2a–NuRD at the promoter. Moreover, knockdown of PRMT5 resulted in a significant increase in γ-globin gene expression in both HUDEP-2 and CD34^+^ progenitor–derived primary erythroid cells, but resulted in only a minimal further increase in MBD2KO HUDEP-2 cells. Thus, PRMT5 occupancy and the majority of its silencing effect are dependent on the presence of MBD2a–NuRD at the promoter. PRMT5 is known to symmetrically methylate arginine in histones H3 and H4 to facilitate inactive chromatin formation (47, 48). Consistent with its role in HBG gene silencing through reinforcement of closed chromatin configuration, nucleosomes bearing H3R8me2s were found to be highly enriched on the promoter in parental, but not in MBD2KO HUDEP-2, cells in which MEP50/PRMT5 no longer occupies the HBG promoter.

In summary, our findings support a model in which the MBD2a–NuRD complex localizes over the methylated proximal HBG promoter facilitating its preferential interaction with BCL11A to form a large repressive complex containing PRMT5. In concert, this complex is required to position and maintain a nucleosome over the promoter which in turn is required for stable competition of the major repressor factor BCL11A over the key activator factor NF-Y to enforce gene silencing. Although not directly tested, the loss of MBD2 likely increases the interaction of the locus control region with the HBG promoters, as does loss of BCL11A. This model helps resolve the question of how CpG methylation at the promoter participates in silencing. As proposed here, it increases the probability of the reader of CpG methylation, MBD2a, localizing there and interacting with the major silencing factor, BCL11A. Recent elegant studies have shown that acute loss of BCL11A results in the activation of γ-globin gene expression without affecting the CpG methylation status at the promoter (49). These findings are consistent with the idea that BCL11A is required for stable occupancy of the MBD2a–NuRD complex at the promoter, since CpG methylation requires a reader to execute repression. Thus, loss of MBD2a at the promoter eliminates the repressive effect of DNA methylation, as we show here. We have reported similar findings in breast cancer cell lines in which MBD2 depletion results in transcriptional activation of silenced, methylated tumor suppressor genes without loss of corresponding promoter methylation (50). Overall, these results suggest a model in which DNA methylation and MBD2a play an active rather than a merely passive role in the occupancy and repressive activity of a BCL11A–MBD2–NuRD complex at the HBG promoter as depicted in the model in Fig. 4*E*. We and others have previously shown that depletion of the components of or disruption of the interactions within the MBD2–NuRD complex results in high levels of HbF expression in adult human erythroid cells (8, 10, 11). The model presented here suggests additional interfaces within this large repressor complex, the disruption of which could be exploited to relieve silencing of HbF expression. This model is testable with a combination of structure-based and gene-editing approaches in primary human erythroid cells in culture and in recently described in vivo models of human CD34+ hematopoietic progenitor cell transplantation in immunocompromised mice (51).

As the intact multicomponent and specific repressor complex described here appears to be essential for complete HbF silencing in adult erythroid cells, continued efforts to identify disruptors of this complex as potential small-molecule therapeutics for sickle cell anemia and β-thalassemia seem justified.

## Materials and Methods

Detailed information about antibodies and other reagents and plasmids used in these studies are provided in *SI Appendix, Materials*.

### Isolation and Maturation of Human CD34^+^ Cells.

Human CD34^+^ cells were purified from deidentified apheresis units discarded by the VCU Bone Marrow Transplant Unit, Institutional Review Board, Exemption 4. CD34^+^ cells were isolated using the EasySep Human CD34–Positive Selection Kit (StemCell Technologies) as described previously (10). Erythroid differentiation and maturation protocols were followed as described previously (52) and monitored by measuring expression of the erythroid lineage markers CD235a and CD71 via Taqman qPCR assay with the probes listed in *SI Appendix*, Table S4.

## Human CD34^+^ Cell Culture and Differentiation.

After isolation, human CD34^+^ cells were initially cultured for expansion in StemSpan SFEM II supplemented as described (53). Cells were differentiated utilizing a three-phase culture protocol. The base culture medium was composited of Iscove’s Modified Dulbecco’s Medium (IMDM), 2% human peripheral blood plasma, 3% human AB serum, 200 μg/mL HTF, 3 IU/mL heparin, and 10 μg/mL insulin. In the first phase (day 0 to day 6), CD34^+^ cells at a concentration of 10^5^ cell/mL were cultured in the presence of 10 ng/mL SCF, 1 ng/mL IL-3, and 3 IU/mL EPO. In the second phase (day 7 to day 11), IL-3 was omitted from the culture medium. In the third phase, SCF was omitted from day 16 to day 21. The cell concentration was adjusted to 10^6^ cell/mL on day 11 and to 5 × 10^6^ cell/mL on day 15. The cells were cultured at 37 °C in the presence of 5% CO_2_.

### HUDEP-2 Cell Culture and Differentiation.

HUDEP-2 cells were maintained and expanded by the method described previously (10, 16) and were kindly provided by Yukio Nakamura. Details of the method are provided in *SI Appendix*, *Methods*.

### Lentiviral Preparation and Transduction.

Lentivirus constructs were packaged into 293T cells and collected for transduction in HUDEP-2 or CD34+ progenitor primary cells as previously described (10). Detailed methods are provided in *SI Appendix*, *Methods*.

### Lentiviral-Mediated “Add-Back” of MBD2a and MBD2b in MBD2 Null Cells.

pLV203 vectors containing sequences encoding MBD2sgR-TAPtag or MBD2b-TAPtag were packaged as described previously (10) and used to infect MBD2KO HUDEP-2 cells. Translationally silent mutations were introduced into the MBD2 expression constructs to confer resistance to MBD2 CRISPR/Cas9 sgRNA. The assessment of exogenous MBD2 expression in HUDEP-2 MBD2KO cells was carried out by western immunoblotting five d post lentiviral infection as described (7).

### ChIP-qPCR Assay.

Chromatin immunoprecipitation-qPCR assays were performed as described (54) with minor modifications. Primers used to detect MBD2 enrichment at the globin promoter regions are included in *SI Appendix*, Table S5, and were designed to generate PCR products of ~100 bp fragment. The quality of ChIP was verified by real-time PCR. Details of the method are provided in *SI Appendix*, *Methods*.

### ATAC-seq Assay.

ATAC-seq was performed as previously described (55). A total of 50,000 cells of expansion-phase HUDEP-2 or derived cell lines were collected and permeabilized with 50 μL ice-cold lysis buffer for 3 min. The transposition reaction was carried out at 37 °C for 30 min in 50 μL volume containing 25 μL 2× TD buffer and 2.5 μL Tn5 Transpose enzyme (Illumina). The resulting libraries were purified using AMPure XP beads (Beckman Coulter) and quantified with Qubit fluorometer and bioanalyzer, and then sequenced in NextSeq 500 platform 76 bp single-read sequencing. Details of the method and ATAC-seq data analysis method are provided in *SI Appendix*, *Methods*.

### NOMe-seq.

Nuclei from 250,000 HUDEP-2 cells were isolated as previously described (56). Thereafter, nuclei were incubated with 200 units of GpC methyltransferase (M.CviPI) and S-Adenosyl methionine (SAM) for 7.5 min at 37 °C followed by a boost with an additional 100 units of M.CviPI and SAM for 7.5 min. The reaction was stopped, DNA was extracted, and bisulfite-converted to distinguish methylated from unmethylated Cs. For individual regions of interest, primers were designed that do not contain any CpG or GpC dinucleotides. PCR was performed, followed by TA cloning and Sanger sequencing. Sequences of PCR primers are listed in *SI Appendix*, Table S5.

### Genome Editing of the MBD2 Y178 Site.

The sequence of adenine base editors NG-ABE8e was cloned into lentiCRISPRv2 to generate a lenti-NG-ABE8e lentiviral vector. The oligos of gRNA sequence targeting the MBD2 Y178 codon were synthesized by IDT. After annealing into dsDNA, the DNA fragment was ligated with lenti-NG-ABE8e vector, followed by lentiviral preparation and transduction. Infected HUDEP-2 cells were selected by puromycin for 3 d post transduction. DNA was extracted for editing efficiency testing by PCR and Sanger sequencing. The selected stable transfected cells were seeded in a 96-well plate for single-colony growth. The single-colony-cloned cells were tested by the same PCR/sequencing method to determine editing efficacy. Both edited clones and unedited clones were differentiated in parallel for 3 d and mRNA harvested for qPCR to quantitate globin gene expression.

### Unbiased Proteomic Assay.

The gel bands from control and MBD2b-TAPtag and MBD2-TAPtag pulldowns containing all proteins from each sample were excised from the gel, cut into equal-sized cubes (approximately 1 mm), and transferred to a siliconized Eppendorf tube. The detailed method for sample preparation is provided in *SI Appendix*, *Methods*.

Samples were analyzed using the LC–MS system which consisted of a Thermo Electron Q-Exactive HF-X mass spectrometer with an Easyspray Ion source connected to an Acclaim PepMap 75 µm × 2 cm nanoviper C18 3 µm × 100 Å precolumn in series with an Acclaim PepMap RSLC 75 µm × 50 cm C18 2 µm bead size (Thermo Scientific). The data were analyzed by database searching using the Sequest HT search algorithm using a custom human database downloaded from Swiss Pro. The following variable modifications were considered: oxidized methionine (+16 m/z) and carbamidomethyl cysteine (+57 m/z). Details of the LC–MS methodology and proteomics data analysis are provided in *SI Appendix*, *Methods*.

## Supplementary Material

Appendix 01 (PDF)

Dataset S01 (XLSX)

## Data Availability

The primary proteomics peptide data is presented in *SI Appendix*, TableS3. Raw and processed ATAC-seq data are available on the NCBI Gene Expression Omnibus at the following URL: https://www.ncbi.nlm.nih.gov/geo/query/acc.cgi?acc=GSE224834, accession number GSE224834 (reviewer’s access token avupcswkxtiztol) (57).
