# Supplementary material for: MBD2a–NuRD binds to the methylated γ-globin gene promoter and uniquely forms a complex required for silencing of HbF expression
Source: Proc Natl Acad Sci U S A. 2023 Jun 12;120(25):e2302254120. doi: 10.1073/pnas.2302254120 (PMC10288633; doi:10.1073/pnas.2302254120)
Supplement: Supplementary file 1 — Appendix 01 (PDF) [file pnas.2302254120.sapp.pdf]

PNAS

Supporting Information for

MBD2a-NuRD Binds to the Methylated γ-Globin Gene Promoter and Uniquely Forms a Complex Required for Silencing of HbF Expression

Shengzhe Shang[a, 1], Xia Li[a, b, 1], Alexander Azzo[c, d], Tin Truong[a], Mikhail Dozmorov[e], Charles Lyons[a], Asit K. Manna[f], David C. Williams, Jr. [f] and Gordon D. Ginder [a, b, g, *]
[a]Massey Cancer Center, Virginia Commonwealth University, Richmond, VA 23060; [b]Department of Human and Molecular Genetics, Virginia Commonwealth University, Richmond, VA 23060; [c]Center for Clinical and Translational Research, PhD Program in Cancer and Molecular Medicine, Virginia Commonwealth University, Richmond, VA 23060; [d]MD-PhD Program, Virginia Commonwealth University, Richmond, VA 23060; [e]Department of Biostatistics, Virginia Commonwealth University, Richmond, VA 23060; [f]Department of Pathology and Laboratory Medicine, University of North Carolina, Chapel Hill; [g]Department of Internal Medicine, Virginia Commonwealth University, Richmond, VA 23060
[1]S. S. and X. L. contributed equally to this work.
* Corresponding author is Gordon D. Ginder.
Email: gdginder@vcu.edu

This PDF file includes:

Supporting text
SI Appendix Figures S1 to S10
SI Appendix Tables S1 to S6
Legends for Datasets S1
SI References

Other supporting materials for this manuscript include the following:

SI Appendix Datasets S1

Supplementary text: Supplementary Materials and Methods.

Materials

Antibodies and reagents

Antibodies used in this study are listed in *SI Appendix* Table S3. Protein G Dynabeads were from Thermo Fisher. Strep-Tactin magnetic kit, MagStrep "type3" XT beads were from IBA. StemSpan SFEM II were purchased from StemCell Technologies. Recombinant human stem cell factor (SCF) and recombinant human Thrombopoietin (TPO) were purchased from R&D systems. Doxycycline (DOX), Dexamethasone (DEX), recombinant human insulin, heparin and human AB serum were obtained from Sigma Aldrich. Human AB plasma was purchased from Seracare. Recombinant Erythropoietin (EPO) was purchased from Virginia Commonwealth University (VCU) main hospital. Holo-human transferrin (HTF) was purchased from Prospec. Human Flt3-ligand was purchased from PeproTECH.

Plasmids

Knockdown lentiviral plasmids shPRMT5#21 and #46 were purchased from Sigma-Aldrich (TRCN0000176621 and TRCN0000303446). NG-ABE8e base editor was purchased from Addgene (#138491). The packaging plasmid psPAX2 (Addgene #12260) and pMD2.G (Addgene #12259), lentiviral pLV203 and CRISPR plasmid LentiCRISPRv2 were as previously saved in our lab (Addgene #98290).

Methods

HUDEP-2 cell culture and differentiation

HUDEP-2 cells were maintained and expanded in StemSpan SFEM II supplemented with 50 ng/mL SCF, 3 IU/mL EPO, 10^{-6} M of DEX, 1 µg/mL of DOX, 1% L-Glutamine and 2% penicillin/streptomycin (expansion medium). To induce erythroid differentiation, HUDEP-2 cells were cultured for 3 days in IMDM supplemented with 3 IU/mL EPO, 1 µg/mL DOX, 10 µg/mL human recombinant insulin, 3 IU/mL heparin, 0.5 mg/mL of HTF, 5% human AB serum, 1% penicillin/streptomycin, and 1% L-Glutamine (differentiation medium). The cells were cultured at 37°C in the presence of 5% CO_2.

Lentiviral preparation

5 x 10^6 293T cells were seeded in 10 cm culture dishes with 10 mL DMEM supplemented with 10% FBS, 1% HEPES, 1% non-essential amino acid (NEAA), 1% penicillin/streptomycin, and 1% L-Glutamine. 24 hours later, the cells were given fresh media and co-transfected with 8 µg of a lentiviral expression plasmid together with 6 µg psPAX2 and 4 µg pMD2.G using polyethyleneimine (PEI). 12-16 hours post transfection, the media was replaced with 5mL DMEM supplemented with 5% FBS, 1% HEPES, and 1% NEAA. Viral supernatants were collected 48 and 72 hours post transfection, filtered through 0.45 µm filters. Viral supernatants were either used fresh to infect target cells or concentrated by ultracentrifugation, snap frozen, stored at -80°C for future use.

Lentiviral transduction of HUDEP-2 cells and $CD34^+$ cells

HUDEP-2 cells were transduced by centrifugation with lentivirus at 2,800 rpm, 32°C for 90 min with 10 µg/mL polybrene in HUDEP-2 expansion medium without antibiotics. The cells were incubated for 3 hours with virus at 37°C, 5% CO_2 and then transferred to fresh medium for further expansion. For lentiviral infection of $CD34^+$ cells, the cells and viral supernatant along with 8 µg/mL polybrene, were spun at 2,000 rpm for 90 min at room temperature.

ChIP-qPCR assay.

The chromatin immunoprecipitation-qPCR assay was performed as described (1) with minor modifications. Briefly, 3 × 10^7 HUDEP-2 cells (undifferentiated, differentiated only for NF-YA ChIP) or 3 × 10^7 $CD34^+$ cells (differentiated in Phase 1 of 3-phases media for 7 days) were crosslinked with 1% formaldehyde for 10 min at room temperature, quenched by 0.125 M Glycine for 5 min. Cells were washed with ice cold PBS twice and lysed by nuclei extraction buffer. Nuclei was resuspended by lysis buffer three and sonicated in a millitube (Covaris) with Covaris E220 ultrasonicator for 13 min. DNA was sonicated sufficiently to obtain an average fragment size of ~200-500 bp. 1 mL sonicated chromatin was mixed with 1/10 volume of 10% Triton X-100, 33 µL Dynal beads protein G (Thermo Fisher Scientific) and 3 µg antibody or normal control IgG. After overnight rotating, the beads were washed 5 times with 1 mL wash buffer. To elute and de-crosslink, 210 µL elution buffer was added to the beads and incubated at 65 °C for 30 min. The beads were then spun down and 200 µL of supernatant was transferred to a new tube and incubated at 65 °C overnight. The eluted material was extracted with phenol-chloroform, and DNA was precipitated by adding 800 µL absolute ethanol. DNA was pelleted by centrifuging at 20,000 g for 10 min at 4°C, washed once with 80% ethanol, then air-dried and dissolved with 30 µL 10 mM Tris-HCl, pH 8.0. Primers used to detect MBD2 enrichment at the globin promoter regions are included in *SI Appendix*, Table S5 and were designed to generate PCR products of ~100 bp fragment. The quality of ChIP was verified by real-time PCR.

Western Blot

Protein samples were lysed with RIPA buffer and prepared for Western blot mix by boiling in 1× SDS loading buffer for 5 min. Followed by separation with 10% SDS-PAGE gels electrophoresis. Proteins were then transferred to a PVDF membrane. After transferring the membrane was blocked with 5% nonfat milk for 1 hour and then incubated with primary antibodies overnight at cold room with shaking. Excess antibodies were washed with TBST (50 mM Tris pH 8.0, 150 mM NaCl, 0.1% Tween 20) for 3 times and HRP-conjugated secondary antibodies were incubated for 1 hour at room temperature. After 3 washes with TBST, the membranes were developed with Western Bright ECL HRP substrate (Advansta, San Jose, CA).

Immunoprecipitation

3 x 10^7 HUDEP-2 cells were lysed in 1mL micrococcal nuclease (MNase) digestion buffer (25 mM HEPES-KOH pH 7.6, 100 mM NaCl, 5 mM $MgCl_2$, 3 mM $CaCl_2$, 0.2% NP-40, 10% glycerol and 1X EDTA-free protease inhibitor cocktail (Roche)) and MNase digestion was performed as described previously (2). After centrifugation, the cleared cell lysate was applied to immunoprecipitation by MagStrep "type3" XT beads or Protein G Dynabeads based on manufacture's protocols. Samples were then boiled in 1× SDS loading buffer for 5 min to denature all proteins and separated with 10% SDS-PAGE gels electrophoresis.

qRT-PCR

Real time PCR was performed using TaqMan primers and carboxyfluorescein (FAM) labeled probe sets from Thermo Fisher or custom probe-primer sets as described previously (3). Primer and probe sequences are listed in *SI Appendix*, Table S5. Target gene expression was normalized to cyclophilin A (PPIA) and analyzed using the $2^{-\Delta\Delta CT}$ relative quantification method.

ATAC-seq assay

ATAC-seq was performed as previously described (4). 50,000 cells of expansion phase HUDEP-2 or derived cell lines were collected, and permeabilized with 50 µL ice cold lysis buffer (10 mM Tris-HCL pH 7.5, 10 mM NaCl, 3 mM $MgCl_2$, 0.1% NP-40, 0.1% Tween-20, 0.01% Digitonin) for 3 min. The transposition reaction was carried out at 37°C for 30 minutes in 50 µL volume containing 25

µL 2× TD buffer and 2.5 µL Tn5 Transpose enzyme (Illumina). DNA was purified with Qiagen MinElute PCR purification kit (Qiagen). Library amplification was performed with NEBNext Ultra II Q5® Master Mix (contained in NEBNext® Ultra™ II DNA Library Prep Kit). PCR amplification was carried out as follow: 72°C 5 min, 98°C 30 s, 98°C 10 s, 63°C 30 s, 72°C 1 min, repeat steps 3-5 for another 4-6 cycles, hold at 4°C. The resulting libraries were purified using AMPure XP beads (Beckman Coulter) and quantified with Qubit fluorometer and bioanalyzer, and then sequenced in NextSeq 500 platform 76 bp single read sequencing.

ATAC-seq data analysis

ATAC-Seq was performed in the Genome Sequencing Facility of Greehey Children's Cancer Research Institute at UT Health – San Antonio according to the provider's protocol. Briefly, samples for HUDEP-2 WT and MBD2KO groups were sequenced in triplicates using 75 bp single read sequencing and NextSeq 75HO kit. The data was quality controlled using FastQC v0.11.9. Adapters were trimmed using TrimGalore v0.6.6. Trimmed ATAC-seq reads were aligned to the human reference genome GRCh38/hg38 (http://hgdownload.cse.ucsc.edu/goldenpath /hg38/bigZips/analysisSet/hg38.analysisSet .chroms.tar.gz, accessed on 12/21/2016) using bowtie v.2.4.2. The resulting alignment files were sorted, indexed, and marked for duplicates using samtools v1.15.1. Reads with mapping quality less than 10 were discarded. Peak calling for nucleosome-free regions was performed using MACS2 v2.2.7.1. The bigWig signal files were produced from MACS2 bedGraph files using bedGraphToBigWig v.4.

Expression and purification of MBD2a domains

The MBD of human MBD2a (residues 150-214) was cloned into a modified pET32a (Novagen) vector with N-terminal thioredoxin and hexahistidine tags followed by a Tobacco Etch Virus protease site, as described previously (2). The GR rich region (residues 1-150) and the GR plus MBD regions (residues 1-214) were cloned into the same vector with an additional C-terminal TwinStrep® tag following a short linker (GSGGSSA) and TEV protease site. The resulting vectors were transformed into Rosetta2(DE3) E. coli (Invitrogen), grown in Luria Bertani media at 37°C until an A_{600} ~ 0.5, and induced with 1 mM of isopropyl β-D-1-thiogalactopyranoside (IPTG) overnight at 16°C. The bacteria were harvested by centrifugation, lysed by sonication, and the proteins purified by nickel affinity chromatography. The GR and GR-MBD proteins were further affinity-purified over a Strep-Tactin®XT 4Flow® column to isolate full-length protein. Both the N- and C-terminal affinity tags were removed by cleavage with TEV protease overnight at room temperature. The proteins were then passed back over a nickel column to remove the TEV protease and fusion tags. The proteins were concentrated and further purified by gel filtration (Superdex-75 Increase 10/300, GE Healthcare) before buffer exchanging or dialyzing into phosphate buffered saline (1xPBS: 11.9 mM PO_4, 137 mM NaCl, 2.7 mM KCl, pH 7.4) plus 1 mM tris (2-carboxyethyl) phosphine (TCEP) for all subsequent analyses.

DNA purification for ITC assay

The different methylated and unmethylated oligonucleotides (*SI Appendix*, Table S6) were purchased from Integrated DNA Technologies, annealed, and purified by anion exchange chromatography (Source™ 15Q 4.6/100 PE, GE Healthcare). The purified DNA was dialyzed against 1xPBS + 1 mM TCEP.

Isothermal titration calorimetry

Data were collected on either a MicroCal Auto-iTC200 or Malvern PEAQ-ITC (Malvern Panalytical) at 37 ℃. The different wild-type MBD2a protein constructs (100 µM) were injected into DNA (10 µM) (20 injections of 2 µL, 180 s between each injection) and the resulting trace integrated and fit to a one-to-one binding model with the PEAQ-ITC analysis software. For MBD2-MBD(Y178H)

proteins, additional experiments were collected with higher concentrations of the protein (720 μM) and DNA (~30μM). All protein and DNA samples were in 1xPBS + 1 mM TCEP.

Thermal denaturation

We determined the thermal denaturation midpoint (Tm) for different MBD2 constructs free and bound to DNA by measuring native tryptophan fluorescence on a Prometheus NT.48 differential scanning fluorometer instrument (NanoTemper). The MBD2a-GR (20 μM), MBD2a-MBD (16 μM), and MBD2a-GRMBD (12 μM) proteins were combined with 10% excess DNA in 1xPBS plus 1mM TCEP in a final volume of 30 μL. Tryptophan fluorescence was recorded at both 330 nm and 350 nm from 20 to 95 ℃ with constant heating (1℃/min). The Tm was determined from the peak maximum of the first derivative of the fluorescence intensity ratio (I350/I330), using the NanoTemper software. Each sample was run in duplicate, and both the folding and unfolding curves were analyzed.

NMR spectroscopy

Isotopically labeled protein was generated by growing the bacteria in M9 media using standard approaches and was purified as unlabeled protein. For samples containing DNA, we added a 17 base-pair double-stranded oligonucleotide described previously (5) with the central CpG dinucleotide (highlighted in bold) methylated or unmethylated (GAGGCGCT**CG**GCGGCAG). Data were collected on a Bruker Avance III HD 850 MHz spectrometer equipped with a TCI H-C/N-D 5 mm CryoProbe. All data were processed with NMRPipe (6) and analyzed with CcpNmr Analysis versions 2 and 3 (7, 8).

Unbiased Proteomic assay

The gel bands (Control, MBD2b-TAPtag and MBD2-TAPtag) containing all proteins from each sample were excised from the gel, cut into equal size cubes (approximately 1mm), transferred to a siliconized Eppendorf tube. The gel pieces were washed with 80% ACN (acetonitrile) for 10 min, followed by MSH_2O (mass spectrometry grade water) for 10 min. Gel pieces were then dehydrated with 50% ACN MSH_2O for 5 min, vacuum centrifuged for 20 min. Samples were then rehydrated with 20 mM DTT (Dithiothreitol) in 100mM ABC (ammonium bicarbonate) and incubated for 1 h at 56°C. Followed by 60 mM IAA (Iodoacetamide) in 100mM ABC for 45 min in the dark. The pieces were dehydrated with 50% ACN MSH_2O for 10 min, vacuum centrifuged for 20 min. To rehydrate the gel cubes, samples were incubated for 10 minutes in 30 μl of 0.1% PM (Protease Max Surfactant) (Promega) 12.5 ng/μl Trypsin (Fisher Scientific) in 100 mM ABC. The gel pieces were then overlaid with 50 μl 0.1% PM in 100 mM ABC and allowed to digest for 2 hours at 37°C. The digest solutions were collected into fresh siliconized tubes and centrifuged at 14,000 rpm, 5 min and then transferred to fresh siliconized tubes. 20 μl of each sample were then loaded onto a zip tip c18 cleanup column (Fisher Scientific). The column was then washed with 5% ACN in 95% MSH_2O and the peptides eluted 10 times with 20 μl (80% ACN in 20% MSH_2O). The 200 μl eluate of peptides were then concentrated on a vacuum centrifuge to dry and resuspended into 20 μl 100 mM ABC for subsequent mass spectrometry analysis.

The LC-MS system consisted of a Thermo Electron Q-Exactive HF-X mass spectrometer with an Easyspray Ion source connected to an Acclaim PepMap 75 μm x 2 cm nanoviper C18 3 μm x 100Å pre-column in series with an Acclaim PepMap RSLC 75 μm x 50 cm C18 2 μm bead size (Thermo Scientific). 4 μl of the 20 μl ziptipped peptides from each sample Control, MBD2a-TAPtag and MBD2b-TAPtag were injected onto the column above. Peptides were eluted from the column with an acetonitrile/0.1% formic acid gradient at a flow rate of 0.3 μl/min over 1.6 h. The nanospray ion source was operated at 1.9kV. The digest was analyzed using the rapid switching capability of the instrument thus acquiring a full scan mass spectrum to determine peptide molecular weights followed by product spectra (15 High Energy C-trap Dissociation HCD spectra). This mode of analysis produces approximately 50,000 MS/MS spectra of ions ranging in abundance over several orders of magnitude.The data were analyzed by database searching using the Sequest HT search algorithm using a custom human database downloaded from Swiss Pro. The following variable

modifications were considered oxidized Methionine (+16m/z) and Carbamidomethyl Cysteine (+57 m/z).

Normalization and differential analysis of proteomics data

Proteomics measurements included samples expressing MBD2a-TAPtag or MBD2b-TAPtag which contain Strep-Tactin tag and flag tag, and the negative control from cell lysate which doesn't express a TAPtag. The MBD2-TAPtag protein was the highest detected in both groups and not in the negative control sample. The negative control measurements were subtracted from the MBD2a and MBD2b expressing sample measurements. Proteins detected at or below 0 levels were removed. The mean-difference (Bland-Altman) plot between the MBD2a and MBD2b expressing sample measurements was used to normalize the data with Loess regression (remove nonlinear biases). Differential proteins were defined as those showing absolute differences larger than 3 standard deviations of the difference distribution. The analysis was performed using R statistical computing environment v.4.0.0.

Liquid chromatography mass spectrometry

Liquid chromatography mass spectrometry was performed using a Qexactive HF-X instrument with a c4 easy spray column. 2.6 µm 150 A 75 µm x 15 cm. Protein mass determination was manually calculated from the charge distribution of the protein. The differentiated cell samples were lysed with MilliQ water supplemented with protease inhibitors, followed by centrifugation at room temperature at 10,000 g for 5 min. 20 µL of protein sample was isolated and eluted on a C4 ziptip into 70 µL. The 70 µL were reduced to 10 µL in a Speedvac and diluted to final volume of 20 µL.

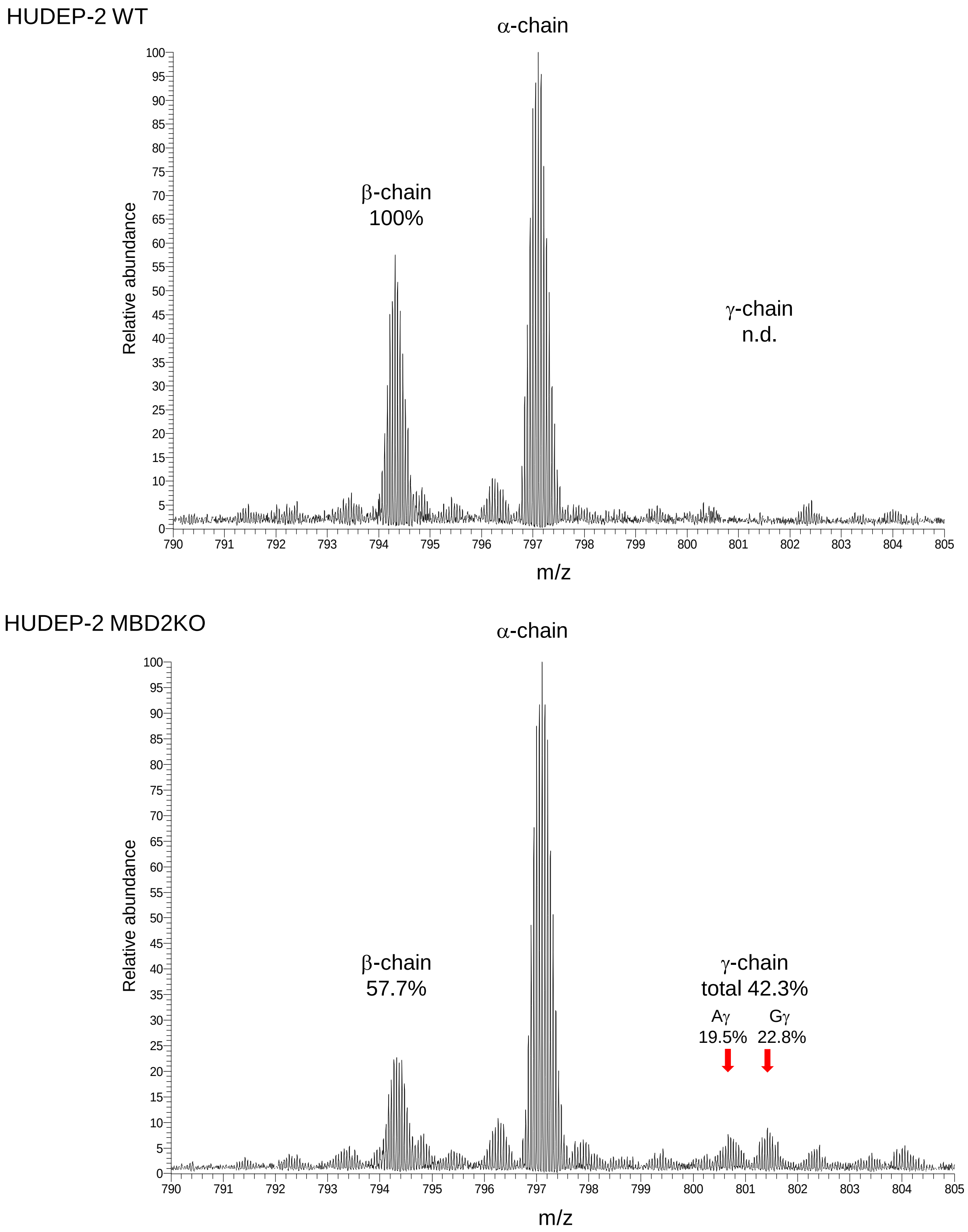

Fig. S1. LC-MS quantitation of globin chains showing the relative percent of total globin chains in parental (Left) and MBD2KO (right) HUDEP-2 cells. Aγ and Gγ, indicated by red arrows, are γ-globin proteins encoded by HBG1 and HBG2 genes respectively.

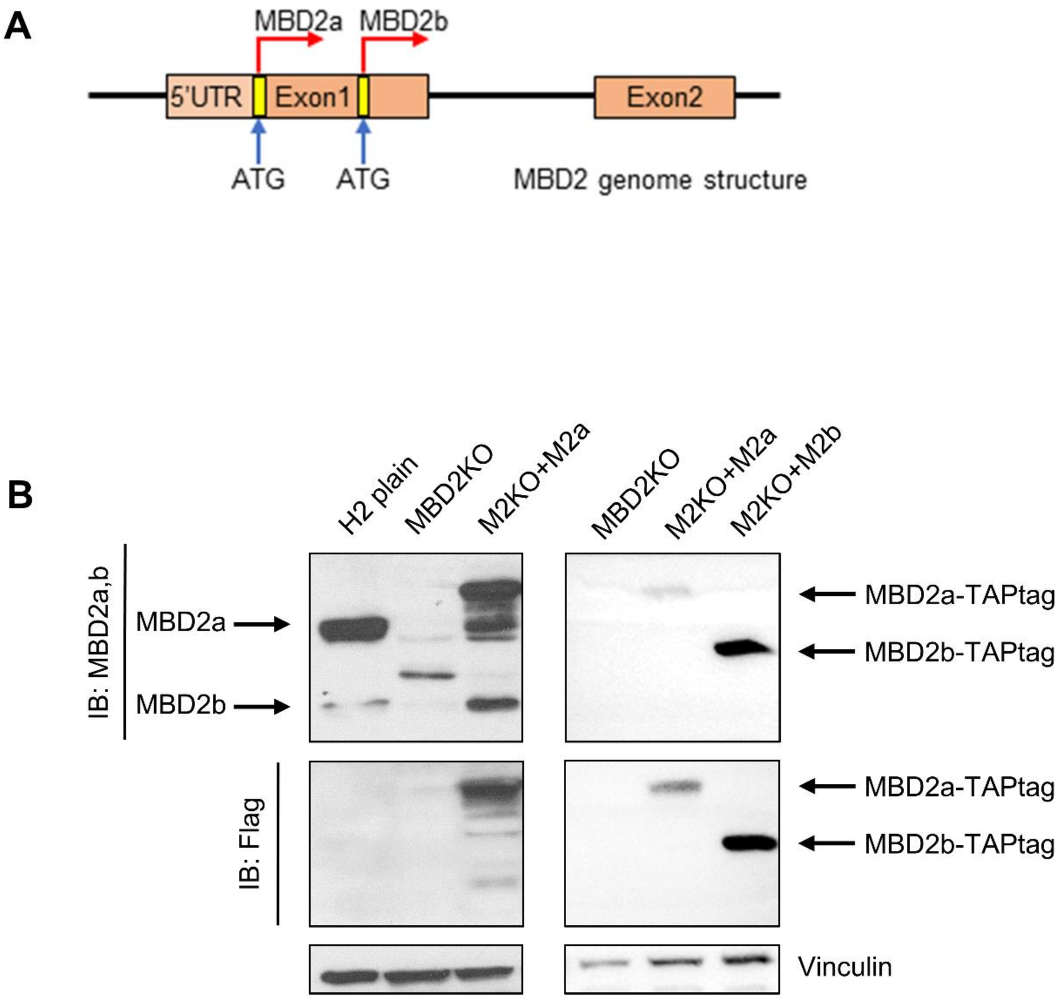

Fig. S2. MBD2a, b gene structure and Western blot of TAPtagged MBD2a and MBD2b isoform proteins. (A) Schematic diagram of MBD2 exon1 in genomic DNA depicting the alternate ATG start codon. (*B*) Western blot showing the sizes and amounts of the endogenous MBD2a, MBD2b and expressed MBD2a-TAPtag as shown in left panel. MBD2a-TAPtag and MBD2b-TAPtag proteins in MBD2 add back MBD2 knockout HUDEP-2 cells from a separate Western blot assay using the same cell populations are shown in the right panel. The band in the MBD2KO/MBD2a addback lane in the left panel that migrates faster than the TAPtag MBD2a addback protein and slower than the endogenous MBD2a in parental HUDEP-2 is an artifact that sometimes appears with the antibody used.

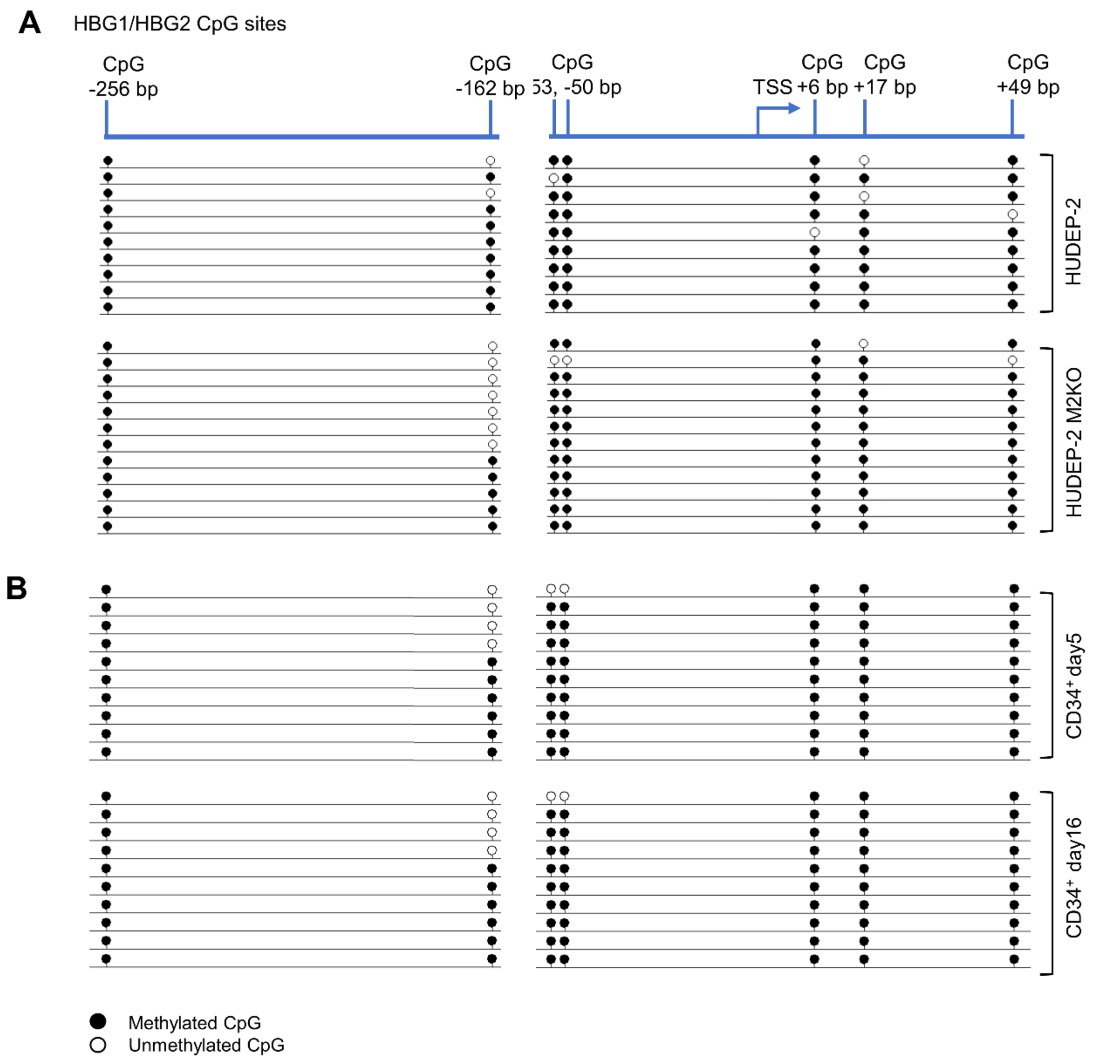

Fig. S3. Methylation of CpG sites in the HBG promoter in parental and MBD2KO HUDEP-2 cells and CD34^{+} progenitor-derived primary erythroid cells. (*A*) Representation of bisulfite DNA methylation assay results showing the same high level of methylation of the CpG sites in the HBG promoter in both parental and MBD2KO HUDEP-2 cells. (*B*) Representation of the results of the DNA methylation assay showing no significant change of methylation at the CpG sites during successive days of erythroid differentiation of primary CD34^{+} progenitor cells.

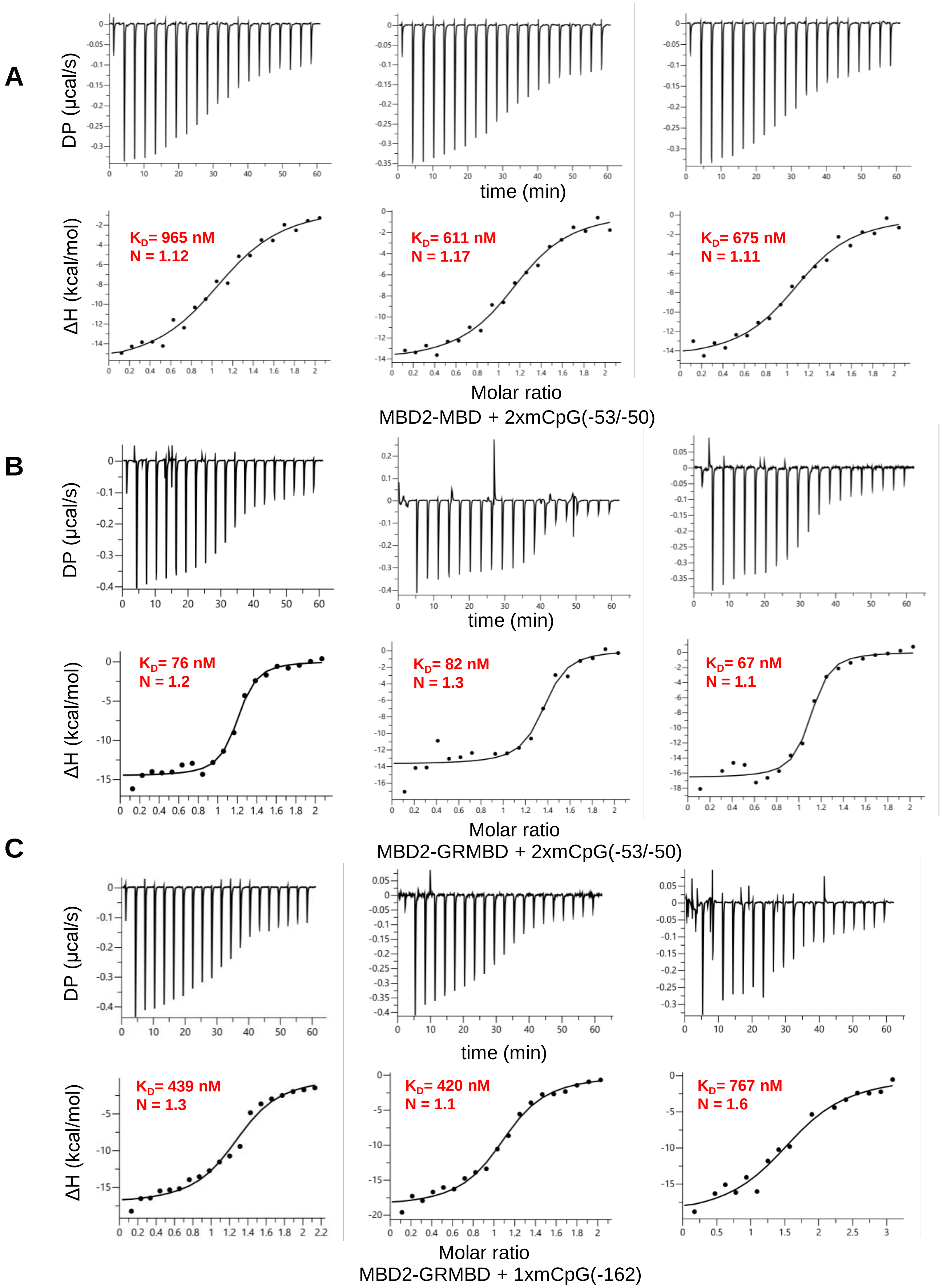

Fig. S4. Protein and methylated DNA binding affinity analysis. All replicate ITC titrations and fits are shown for MBD2-MBD and MBD2-GRMBD binding to the indicated methylated DNA.

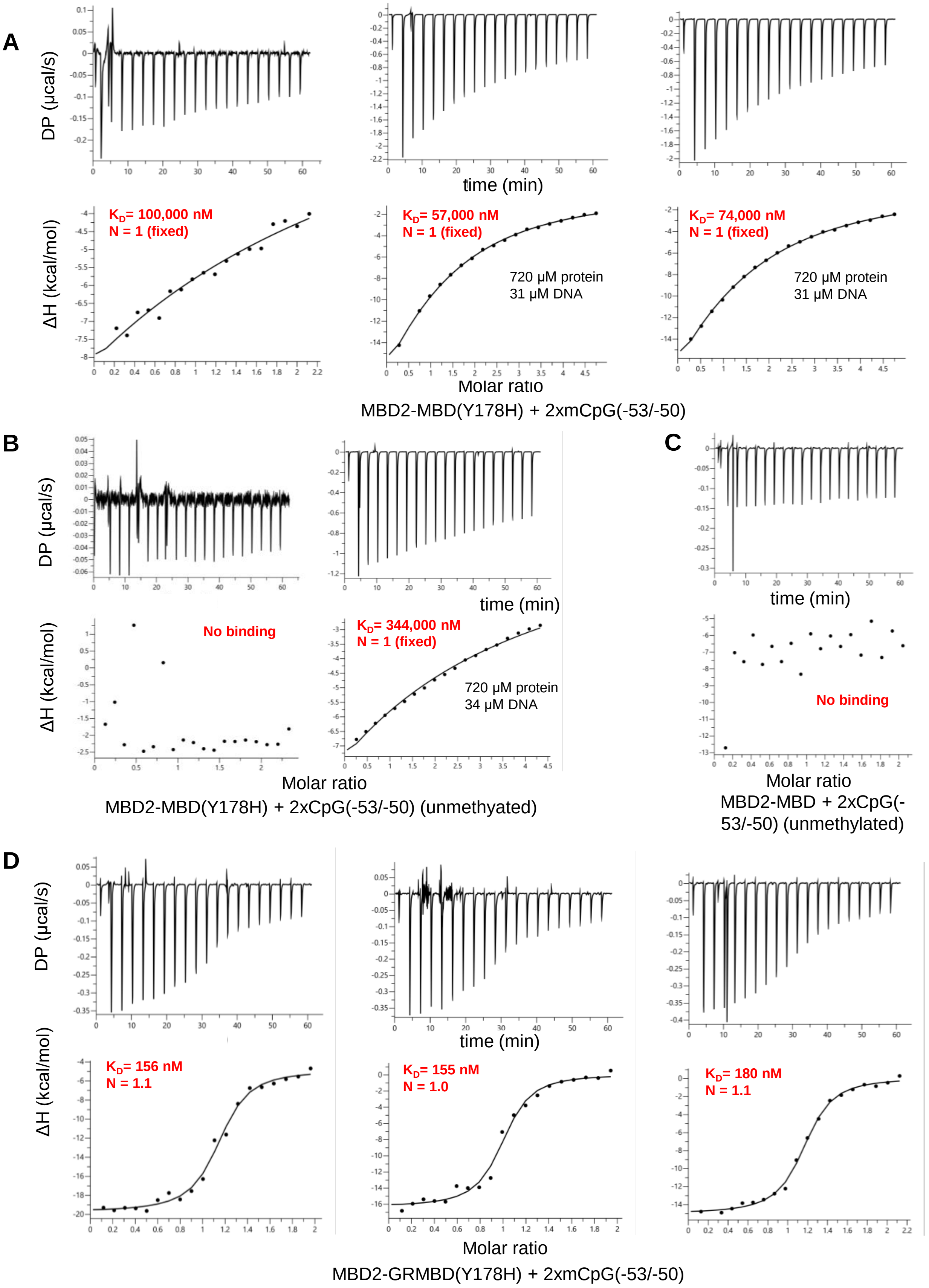

Fig. S5. Protein and methylated or unmethylated DNA binding affinity analysis. All replicate ITC titrations and fits are shown for MBD2-MBD and MBD2-GRMBD(Y178H) binding to the indicated methylated or unmethylated DNA.

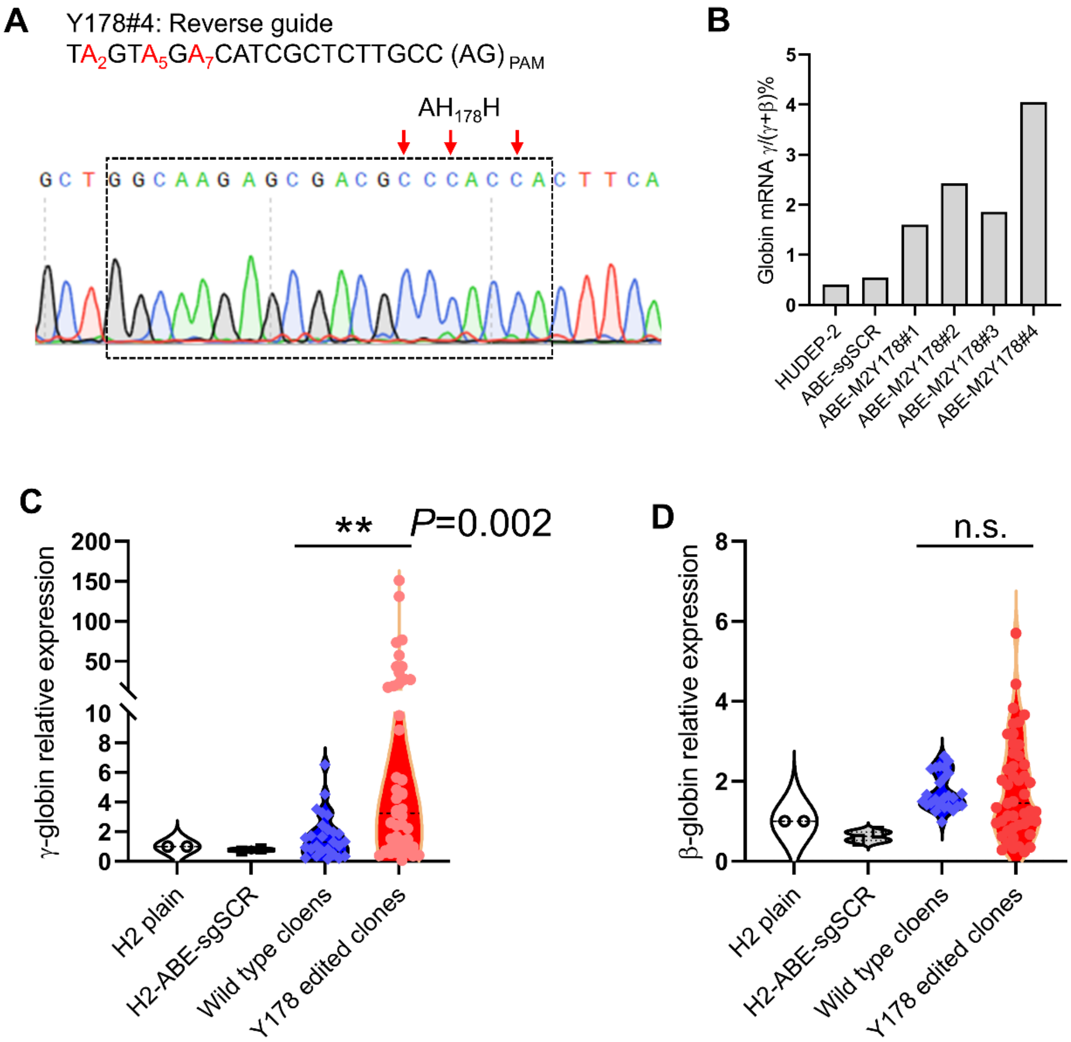

Fig. S6. Adenine base editor introduced the Y178H mutation in endogenous MBD2 in HUDEP-2 cells. (*A*) Sanger sequencing result showing the specific adenine base editing to introduce the Y178H mutation in endogenous MBD2 genomic DNA. The Y178#4 guide sequence as shown above. The black dotted frame shows the guide range. In edited clones, the Y178 is changed to H, V177 is potentially changed to A and Y179 is potentially changed to H. The sequencing result is representative of AHH mutation. The potential edited bases are indicated by red arrows. (*B*) Q-PCR results showing the γ/ (γ+β) mRNA ratio in ABE8e-scramble control transfected cells (sgSCR) and the different guides of ABE8e-MBD2 Y178 edited bulk HUDEP-2 cells. The #4 guide bulk cells were used for further single colony culture and analysis. (*C*) Q-PCR results showing the γ-globin mRNA level and (*D*) the β-globin mRNA level in ABE8e-sgSCR cells and ABE8e-MBD2 Y178 edited single colonies. The *P* value was calculated by Welch's unpaired t-test. Blue dots represent the scramble guide clones without editing at MBD2Y178 (*N*=26), red dots represent the clones with specific editing at MBD2Y178H codon (*N*=47). **P*<0.05, ** *P*<0.01.

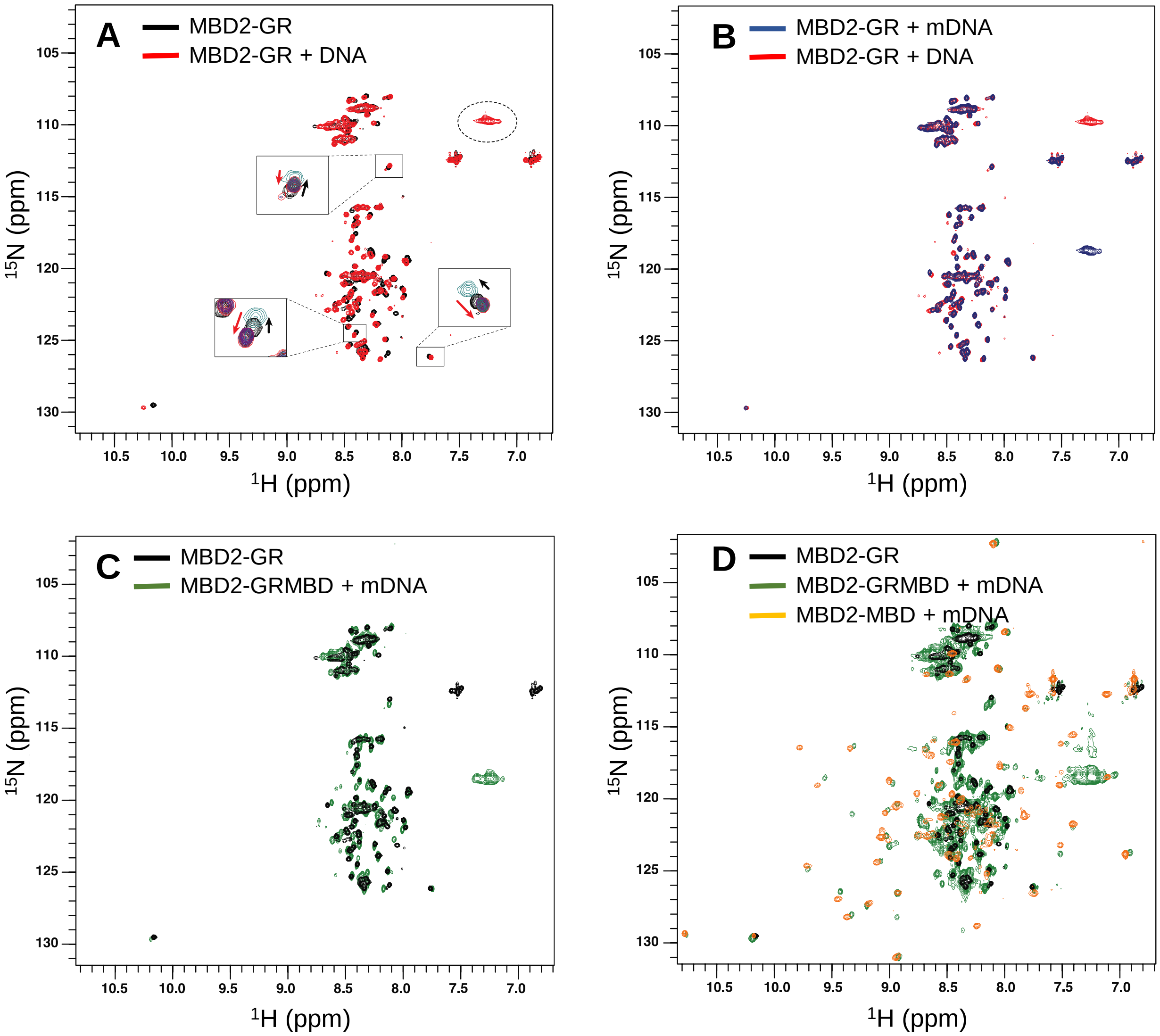

Supplement FigureS7. NMR analyses of MBD2-GR structure and binding. (*A*) 2D ^{15}N-HSQC spectra of ^{15}N-MBD2-GR (80 μM) in isolation (black) and bound to excess unmethylated DNA (480 μM, red). Insets show chemical shift changes over a range of DNA concentrations for select resonances: [DNA] = 40 μM (teal), 80 μM (purple), 160 μM (green), 240 μM (navy), 320 μM (maroon), and 400 μM (sky blue). Arrows highlight the direction of chemical shift changes for DNA/protein molar ratios = 0.5 (black) and ≥1.0 (red). The dashed circle highlights resonances for arginine Nε, which are observed only in the presence of DNA. (*B*) 2D ^{15}N-HSQC spectra of ^{15}N-MBD2-GR in the presence of unmethylated (red) and methylated (blue) DNA are nearly identical. Of note, the Nε resonances show an apparent chemical shift change in ^{15}N due to a difference in sweep width. (*C*) 2D ^{15}N-HSQC spectra of the isolated MBD2-GR (black) and MBD2-GRMBD in the presence of methylated DNA (green) are plotted at a high contour level. This comparison shows that resonances from the MBD2-GR region remain sharp and lack chemical shift dispersion even when bound to DNA through the MBD. (*D*) Plotting the 2D ^{15}N-HSQC spectrum of MBD2-GRMBD at a lower contour level (green) shows well-dispersed peaks with chemical shifts similar to the isolated MBD2-MBD (orange) when bound to methylated DNA.

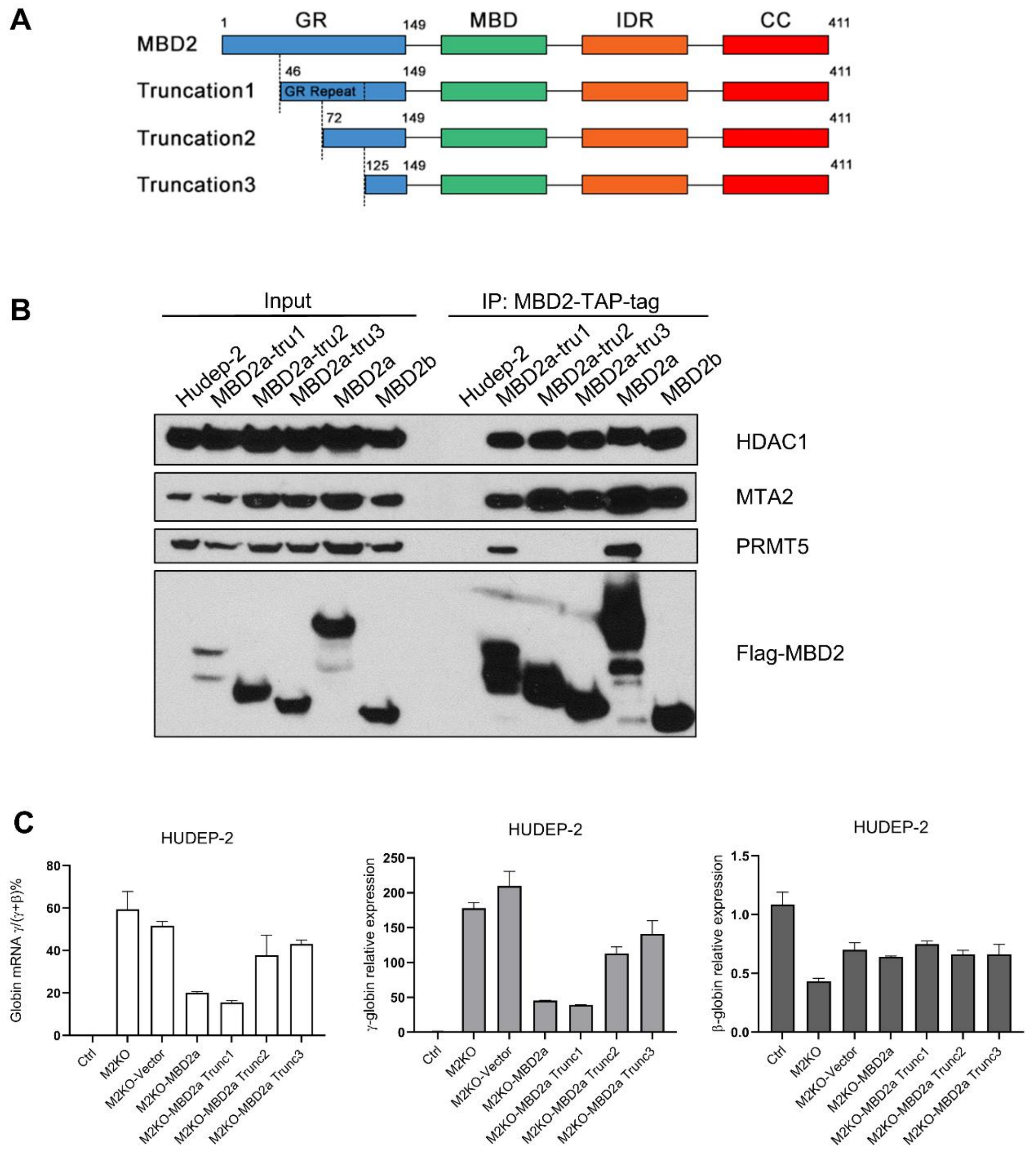

Fig. S8. MBD2 requires the N-terminal 72 aa for the recruitment of PRMT5 and silencing. (*A*) Schematic representation of MBD2 GR domain truncation mutants. (*B*) Western blot showing that the truncation mutant lacking first 72 amino acids of the GR domain associates with core NuRD components but not PRMT5. (*C*) Results of Q-PCR assays of γ- and β-globin mRNA expression with addback of different MBD2 truncation mutants in MBD2KO HUDEP-2 cells showing that the first 72 amino acids of GR domain are required for silencing.

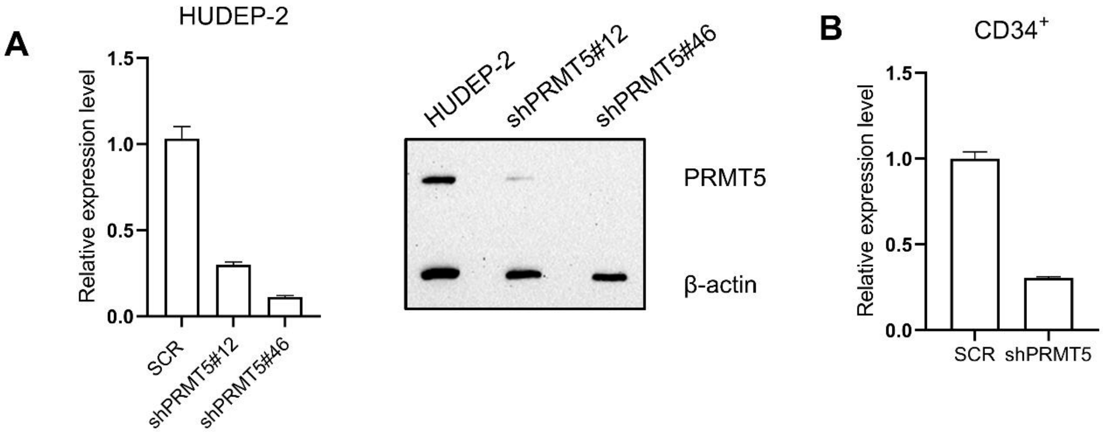

Fig. S9. PRMT5 knockdown in HUDEP-2 and CD34^{+} cells. (*A*) PRMT5 knockdown efficiency assessment by Q-PCR and Western blot assays in HUDEP-2 cells. (*B*) PRMT5 knockdown efficiency assay results by Q-PCR in CD34^{+} cells.

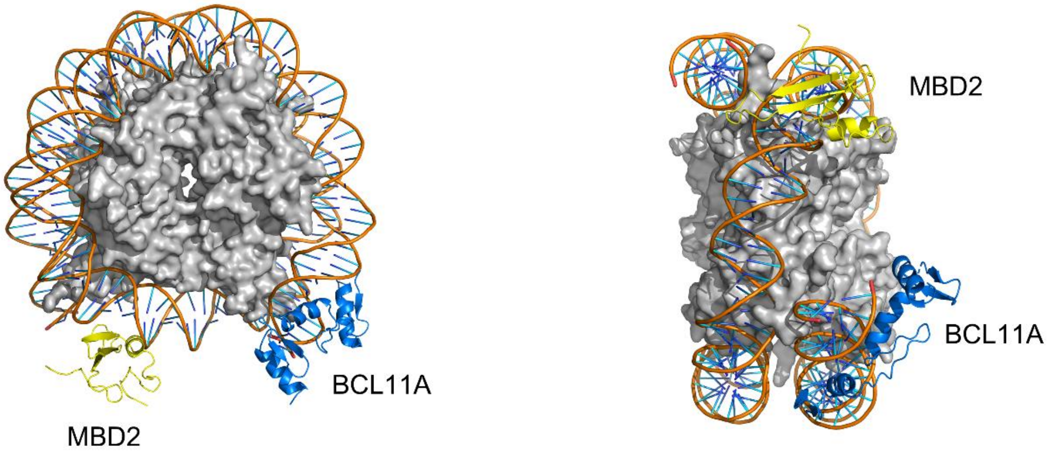

Fig. S10. Molecular model of MBD2 and BCL11A localization with respect to the nucleosome positioned at the proximal HBG promoter. Based on our localization of nucleosomes in the proximal gamma promoter, the 2xmCpG (-53/-50) binding site maps to the dyad of the nucleosome. Shown are two views of MBD2 MBD (yellow, PDBID: 2ky8) bound to the dyad of the nucleosome core particle (nucleosome DNA depicted as a cartoon diagram and the octamer as a gray surface, PDBID: 3lz0). Based on this position, the distal BCL11A binding site maps to the beginning of the linker region, such that the BCL11A DNA binding domain (blue) is near the MBD2 MBD.

Table S1. ChIP-qPCR original data

HUDEP-2 cells	Wild type		MBD2KO	
**MBD2 ChIP**	**HBG**	**HBB**	**HBG**	**HBB**
Relative enrichment of repeat 1	14.70	1.08	0.65	0.64
Relative enrichment of repeat 2	15.83	1.22	0.66	0.50

$CD34^+$ cells	HBG	HBB
Relative enrichment technical repeat 1	17.57	3.39
Relative enrichment technical repeat 2	33.83	3.25
Relative enrichment technical repeat 3	26.75	4.27
standard deviation	8.15	0.56

HUDEP-2 cells	Wild type		MBD2KO	
**NF-YA ChIP**	**HBG**	**HBB**	**HBG**	**HBB**
Relative enrichment of repeat 1	0.15	2.42	20.41	14.67
Relative enrichment of repeat 2	1.08	4.07	3.16	8.14

HUDEP-2 cells	Wild type		MBD2KO	
**MEP50 ChIP**	**HBG**	**HBB**	**HBG**	**HBB**
Relative enrichment of repeat 1	24.76	1.84	1.33	1.21
Relative enrichment of repeat 2	28.10	1.58	2.80	5.59

HUDEP-2 cells	Wild type			MBD2KO		
**H3R8me2s ChIP**	**HBG**	**HBB**	**GAPDH**	**HBG**	**HBB**	**GAPDH**
Relative enrichment of repeat 1	5.14	4.79	18.19	1.63	2.17	5.82
Relative enrichment of repeat 2	16.53	9.07	14.74	0.75	3.12	8.01

.

Table S2. Globin mRNA expression level in differentiated wild type clones and Y178 edited clones

WT clone ID	Globin mRNA γ/(γ+β)%	γ-globin mRNA expression level	β-globin mRNA expression level	Genotype
1	0.16	0.83	2.09	VYY
2	0.05	0.16	1.37	VYY
3	1.85	6.54	1.43	VYY
4	0.43	1.72	1.64	VYY
5	1.30	4.54	1.41	VYY
6	0.63	2.13	1.38	VYY
8	0.39	1.32	1.40	VYY
11	0.27	1.03	1.59	VYY
12	0.55	1.67	1.26	VYY
19	0.75	3.12	1.69	VYY
20	0.22	0.81	1.50	VYY
23	0.29	1.87	2.63	VYY
24	0.04	0.22	2.38	VYY
28	0.44	1.05	0.98	VYY
29	0.11	0.33	1.25	VYY
31	0.12	0.70	2.50	VYY
34	0.29	1.58	2.20	VYY
35	0.52	1.98	1.55	VYY
37	0.04	0.21	1.96	VYY
38	0.61	3.52	2.35	VYY
41	0.10	0.36	1.46	VYY
42	1.00	3.27	1.34	VYY
46	0.08	0.47	2.31	VYY
53	0.64	2.45	1.59	VYY
59	0.23	0.92	1.67	VYY
60	0.23	1.30	2.35	VYY

Y178 edited lone ID	Globin mRNA γ/(γ+β)%	γ-globin mRNA expression level	β-globin mRNA expression level	Genotype
2'	0.49	0.96	0.94	V/A H Y/H
3'	1.44	1.38	0.45	AHH
4'	0.07	0.06	0.35	AH Y/H
7	0.43	2.42	2.30	AH Y/H
9	16.64	131.10	2.70	VHY/H
9'	2.51	4.53	0.62	V/A H Y/H
10'	1.36	0.86	0.24	AHH
10	0.20	0.61	1.24	VHY/H

12	5.84	1.67	1.26	VHY
13	18.49	73.58	1.34	AHH
14	2.05	22.52	4.43	V/A H Y/H
14'	0.83	0.72	0.37	AH Y/H
15'	4.08	5.67	1.14	V/A H Y/H
15	1.86	4.61	1.00	V/A Y/H Y
16	34.24	151.31	1.20	V/A H Y
16'	4.13	2.50	0.32	AH Y/H
17'	1.20	0.60	0.28	VHY
17	0.54	4.84	3.66	AHH
18	0.16	1.52	3.83	V/A H Y/H
21	14.97	43.35	1.01	AHY
22	3.70	27.25	2.92	VHY
22'	2.04	3.54	0.67	VHY
25	17.12	57.44	1.14	AHH
26'	0.20	0.39	0.91	VHY
26	0.08	0.37	1.97	AHH
27	0.45	2.22	2.03	AH Y/H
27'	0.36	0.60	0.59	VHY
30	1.69	8.88	2.13	AH Y/H
30'	1.53	5.50	1.50	AHH
32	10.96	43.47	1.45	V/A Y/H Y/H
33	4.10	27.38	2.64	V/A H Y/H
36	2.22	19.18	3.48	VHY
39	0.48	3.43	2.95	AH Y/H
43	3.25	17.34	2.13	AHH
44	16.29	35.82	0.76	AHH
45	0.26	0.53	0.84	VY Y/H
47	0.36	1.48	1.68	AHY
48	0.40	3.07	3.19	AHY
49	0.71	9.84	5.70	AH Y/H
51	24.96	76.84	0.96	AHH
52	0.34	2.61	3.18	VY Y/H
55	0.24	1.12	1.92	VY Y/H
56	0.18	0.90	2.02	VY Y/H
57	0.56	3.26	2.38	AH Y/H
58	0.17	0.41	1.02	VY Y/H
61	0.51	4.24	3.44	AH Y/H
62	0.13	0.79	2.42	AHH

Table S3. Antibodies used in the experiments.

Antibody Name	Catalog	Brand	Experiment
MBD2	M7318	Sigma-Aldrich	Immunoblot, IP
BCL11A	sc-514842	Santa Cruz Biotechnology	Immunoblot
PRMT5	sc-376937	Santa Cruz Biotechnology	Immunoblot
NF-YA	sc-17753X	Santa Cruz Biotechnology	Immunoblot
Vinculin	sc-73614	Santa Cruz Biotechnology	Immunoblot
Flag	F1804	Sigma-Aldrich	Immunoblot
HDAC1	2602	Cell Signaling Technology	Immunoblot
MTA2	ab66051	Abcam	Immunoblot
β-actin	A1978	Sigma-Aldrich	Immunoblot
BCL11A	ab191401	Abcam	IP
PRMT5	ab109451	Abcam	IP
MBD2	ab45027	Abcam	ChIP
NF-YA	ab139402	Abcam	ChIP
MEP50	ab190361	Abcam	ChIP
H3R8me2s	ab130740	Abcam	ChIP
Normal mouse IgG	sc-2025	Santa Cruz Biotechnology	IP, ChIP
Normal rabbit IgG	5127	Cell Signaling Technology	IP, ChIP

Table S4. Taqman primers and probes used in the experiments.

Target	Vendor	Identifier	Forward Primer	Reverse Primer	Probe
Human PPIA	Thermo Fischer	Hs99999904_m1			
Human PRMT5	Thermo Fischer	Hs01047356_m1			
Human TFRC(CD71)	Thermo Fischer	Hs00951083_m1			
Human GYPA(CD235a)	Thermo Fischer	Hs01068072_m1			
Human MBD2	IDT		5'-TTA ACA CAT CTC AAC CCC TCT G	5'-TGT CTG CCA TCA GTG CTT C	/56-FAM/TTG CTG TAC /ZEN/ TCG CTC TTC CTG TTT CC /3IABkFQ/
Human γ-globin	IDT		5'-GTG GAA GAT GCT GGA GGA GAA A	5'-TGC CAT GTG CCT TGA CTT TG	FAM/AGG CTC CTG GTT GTC TAC CCA TGG ACC /BHQ
Human β-globin	IDT		5'-GCA AGG TGA ACG TGG ATG AAG T	5'-TAA CAG CAT CAG GAG TGG ACA GA	FAM/CA GGC TGC TGG TGG TCT ACC CTT GGA CCC/BHQ

Table S5. Sequences of oligonucleotides used in the experiments.

Name	Sequence	Experiment
HBG ChIP F	GGTCCCTGGCTAAACTCCAC	ChIP-qPCR
HBG ChIP R	CCTGGCCTCACTGGATACTC	ChIP-qPCR
HBB ChIP F	TAGAGGGAGGGCTGAGGGTTTG	ChIP-qPCR
HBB ChIP R	CAGGGTGAGGTCTAAGTGATGA	ChIP-qPCR
GAPDH Promoter ChIP F	TCCCCTTCCTGCAGACAGCTCC	ChIP-qPCR
GAPDH Promoter ChIP R	AGGGAGGGCAGCATACCGGG	ChIP-qPCR
HBG1/2 BC 6F	ATTTTTGAATTTTTTAAAAATTGGTATATG	NOMe-Seq
HBG2 BC 8R	ATTCAATCATTCCAATTTTTCTCTAATTTA	NOMe-Seq
HBG2 BC 5F	GGGAAGAATAAATTAGAGAAAAATTGGAAT	NOMe-Seq
HBG1/2 BC 9R	CCTTCCTTCCCTCCCTTATCCTAAT	NOMe-Seq

Table S6. DNA sequences for ITC

DNA	sequence
1xmCpG (-162)	**5'-ATCTCAATGCAAATATCTGTCTGAAA(mC)GGTCCCTGGCTAAACTCCACCCAT-3'**
2xmCpG (-53/-50)	**5'-GGCCAGGGGC(mC)GG(mC)GGCTGGCTAGGGATGAAGAATAAAAG-3'**

SI Appendix Dataset S1 (separate file). Unbiased subtraction proteomic analysis

Peptide enumeration showing the presence of core NuRD components in both MBD2a and MBD2b immunoprecipitated protein samples. EV represents empty vector control.

SI References

1. T. I. Lee, S. E. Johnstone, R. A. Young, Chromatin immunoprecipitation and microarray-based analysis of protein location. *Nat. Protoc.* **1**, 729–748 (2006).

2. M. A. Desai, *et al.*, An intrinsically disordered region of methyl-CpG binding domain protein 2 (MBD2) recruits the histone deacetylase core of the NuRD complex. *Nucleic Acids Res.* **43**, 3100–3113 (2015).

3. M. Amaya, *et al.*, Mi2β-mediated silencing of the fetal γ-globin gene in adult erythroid cells. *Blood, The Journal of the American Society of Hematology* **121**, 3493–3501 (2013).

4. K. H. K. Lab, K. H. Kaestner Lab, S. Shapira, ATAC Sequencing Protocol v1 https:/doi.org/10.17504/protocols.io.bv9mn946.

5. J. M. Cramer, *et al.*, Probing the dynamic distribution of bound states for methylcytosine-binding domains on DNA. *J. Biol. Chem.* **289**, 1294–1302 (2014).

6. F. Delaglio, *et al.*, NMRPipe: a multidimensional spectral processing system based on UNIX pipes. *J. Biomol. NMR* **6**, 277–293 (1995).

7. S. P. Skinner, *et al.*, CcpNmr AnalysisAssign: a flexible platform for integrated NMR analysis. *J. Biomol. NMR* **66**, 111–124 (2016).

8. W. F. Vranken, *et al.*, The CCPN data model for NMR spectroscopy: development of a software pipeline. *Proteins* **59**, 687–696 (2005).
